# Microbiota, Inflammation and Colorectal Cancer

**DOI:** 10.3390/ijms18061310

**Published:** 2017-06-20

**Authors:** Cécily Lucas, Nicolas Barnich, Hang Thi Thu Nguyen

**Affiliations:** M2iSH, UMR 1071 Inserm, University of Clermont Auvergne, INRA USC 2018, Clermont-Ferrand 63001, France; cecily.lucas@uca.fr (C.L.); nicolas.barnich@uca.fr (N.B.)

**Keywords:** colorectal cancer, intestinal microbiota, inflammation, genotoxins, host-pathogen interaction

## Abstract

Colorectal cancer, the fourth leading cause of cancer-related death worldwide, is a multifactorial disease involving genetic, environmental and lifestyle risk factors. In addition, increased evidence has established a role for the intestinal microbiota in the development of colorectal cancer. Indeed, changes in the intestinal microbiota composition in colorectal cancer patients compared to control subjects have been reported. Several bacterial species have been shown to exhibit the pro-inflammatory and pro-carcinogenic properties, which could consequently have an impact on colorectal carcinogenesis. This review will summarize the current knowledge about the potential links between the intestinal microbiota and colorectal cancer, with a focus on the pro-carcinogenic properties of bacterial microbiota such as induction of inflammation, the biosynthesis of genotoxins that interfere with cell cycle regulation and the production of toxic metabolites. Finally, we will describe the potential therapeutic strategies based on intestinal microbiota manipulation for colorectal cancer treatment.

## 1. Introduction

Colorectal cancer (CRC) is the third most common cancer in both males and females with about 1.36 million of new cases per year and the fourth leading cause of cancer-related deaths worldwide with 700,000 deaths per year [1]. 

CRC formation begins with the transformation of the normal epithelium mucosa into hyper-proliferative epithelium. These hyper-proliferative intestinal epithelial cells (IECs) lose their organization and structure and have the ability to form adenomas. Adenomas can then growth and invade the submucosa and become cancerous with the ability to disseminate into the colon [2]. This series of events, called “adenoma-carcinoma sequence”, which leads to CRC, is heterogeneous, and, depending on the molecular alterations during this sequence, different subtypes of CRC have been described. Three major mechanisms of genetic instability have been described in the framework of sporadic CRC: chromosomal instability (CIN), microsatellite instability (MSI) and CpG island methylator phenotype (CIMP). These mechanisms have an impact on the major signaling pathways and lead to the loss of control of cell proliferation, unlimited cell growth and tumor development. 

About 10% of CRC cases are hereditary, and up to 90% are sporadic (without family history or genetic predisposition). Several risk factors for the development of CRC have been identified, including unhealthy behaviors such as physical inactivity, smoking, and red and processed meat as well as alcohol consumption. Some diseases including obesity, diabetes type 2 and inflammatory bowel diseases (IBD) have been also associated with increased risk to develop CRC [3].

It has been proposed that CRC occurrence may also be influenced by the intestinal microbiota which the gut is in constant exposition with. CRC preferentially affects the large intestine, where the bacterial density is largest (10^12^ cells per mL versus ~10^2^ cells per mL in the small intestine) [4]. Several studies have linked a modification of intestinal mucosa-associated microbiota composition in patients with CRC compared to control subjects [5,6,7]. Moreover, in animal models of CRC (genetic or chemical-induced), those bearing the normal intestinal microbiota (conventional animals) develop more tumors than those deprived of the intestinal microbiota (germ-free animals). These observations suggest that intestinal microbiota is a new player in CRC development. Over the last decades, many discoveries have been made to understand the mechanisms by which the intestinal microbiota acts on the development of CRC. The accepted model of bacteria-induced CRC mechanism is based on the enhanced release of toxins produced by bacteria, the decrease of beneficial bacterial-derived metabolites, the disruption of epithelial barrier, the production of pro-carcinogenic compounds and alterations in the intestinal microbiota or dysbiosis; all of these mechanisms lead to an aberrant activation of the immune system with chronic inflammation, increased cellular proliferation and thus increased CRC development [8]. This model of bacteria-host interaction in CRC has helped pave the way to new therapeutic strategies such as supplementation of microbial fermentation products such as short-chain fatty acids (SCFAs), which have anti-inflammatory and anti-carcinogenic effects [9]; direct suppression of bacterial toxin-induced DNA damage and tumorigenesis using small inhibitor molecules [10]; use of prebiotics shown to decrease carcinogen-induced aberrant crypt foci number in vivo [11]; consumption of lactic acid bacteria-containing probiotics, which can prevent DNA damage induced by the mutagenic and carcinogenic heterocyclic amines [12]; and the use of bacteria such as *Bifidobacterium* and *Bacteroides* to enhance anti-tumor immune therapy efficiency and therefore improving tumor control [13,14].

This review will focus on the current knowledge of the contribution of the intestinal microbiota, especially bacteria, to CRC development, and more particularly how it influences the initiation and the progression of CRC via its different pro-carcinogenic effects including the induction of inflammation, the biosynthesis of genotoxins that interfere with cell cycle regulation, the production of toxic metabolites. Finally, we will discuss the potential therapeutic strategies for CRC treatment based on manipulation of intestinal microbiota. 

## 2. Determinant Factors of Colorectal Cancer (CRC)

CRC is the third most commonly diagnosed cancer in males and the second in females, with 1.36 million new cases per year and almost 694,000 deaths in 2012 [1].The risk of developing CRC increases with age. Additional risk factors are inherited genetic factors, lifestyle and some diseases such as obesity, diabetes type 2 and IBD. Only 5–6% of CRC cases involve inherited genetic alterations. It has been shown that having one or two first-degree relatives with CRC is associated, respectively, with 2.26- and 3.76-fold increased risk to develop CRC [15].

The two main forms of hereditary CRC are the Lynch syndrome or non-polyposis colon cancer, which involves mutations in the DNA mismatch repair system, and the familial adenomatous polyposis (FAP), which is caused by germline mutations in the tumor suppressor adenomatous polyposis coli (*Apc*) gene [16]. 

Beside the uncontrollable genetic factor, several lifestyle factors play an important role and are responsible of approximately 90% of CRC occurrence. Indeed, CRC incidence is very inconsistent over the world, with the highest rates in Europe, New Zealand, United States and Australia, and the lowest rates in Africa and South Asia [1]. In 2012, one study showed a large disparity of CRC occurrence depending on socioeconomic status with an increased risk for the lowest socioeconomic status compared to the highest one due to the highest prevalence of adverse health behaviors such as unhealthy diet, alcohol consumption, smoking, obesity and absence of physical activity [17]. Indeed, diet plays an important role in the occurrence of CRC, and it has been estimated to be involved in 30% to 50% of CRC worldwide. Studies have shown that red meat consumption, low fiber, calcium, folic acid and vitamin D diet could enhance the risk to develop CRC [18]. Alcohol consumption has been suspected to be implicated in CRC development, as the compound resulted from the metabolism of alcohol, acetaldehyde, has mutagenic and pro-carcinogenic activities [19]. In addition, it was shown that alcohol consumption enhances the risk to develop CRC in a dose-dependent manner. Indeed, a pooled analysis of eight cohort studies showed that the consumption of 30 g of ethanol per day or greater during a maximum of 6–16 years of study period enhances the risk to develop CRC by 16%, and 45 g of ethanol enhances the risk by 41% [20]. Cigarette smoking also increases the risk to develop CRC in a time and dose-dependent manner. In 2008, a meta-analysis showed that smokers have 18% increased risk to develop CRC compared to never-smokers [21]. 

Obesity is a risk factor of various cancers, including pancreatic, kidney, liver, breast, esophageal, gastric and colorectal cancer and has been estimated to account for 14% of cancer deaths in men and 20% of cancer deaths in women [22]. Recently, a meta-analysis on 9,000,000 participants from different countries showed that the obese category has a risk to develop CRC 1.3 time higher than the normal category [23]. Later, studies have tried to reveal the molecular link between obesity and CRC. Lin and colleagues showed that diet-induced obesity leads to a silencing of the colonic cell surface receptor guanylyl cyclase C due to loss of expression of its paracrine hormone ligand guanylin. The authors showed that the loss of guanylin is associated with epithelial dysfunction, colon endoplasmic reticulum stress and promoted tumorigenesis in mice treated with the carcinogenic agent azoxymethane (AOM) [24]. Other studies have linked the obesity-associated hormone leptin with the occurrence of CRC, as its expression is enhanced in CRC compared to normal colorectal epithelium and colorectal adenomas [25]. In vitro, this adipokine is able to activate the phosphoinositide 3-kinase (PI3K)/protein kinase B (PKB/AKT)/*mammalian target of rapamycin* (mTOR) signaling pathway and therefore enhance proliferation and inhibit apoptosis of the human HCT116 colon cancer cells [26]. The risk to develop CRC is decreased with physical activity practicing [27]. Indeed, people with no or low physical activity have 27% more risk to develop CRC compared to people with physical activity [28]. In people with high physical activity, incidence of CRC is reduced by 40–50% compared to those with little or no physical activity [29]. It has been proposed that physical activity may decrease the risk to develop various cancers including CRC by decreasing central adiposity, influencing sexual and metabolic hormone levels, reducing inflammation and improving immune function [30]. 

Chronic inflammation is one of the major risks of CRC. Patients with IBD, including ulcerative colitis and Crohn’s disease, have a higher risk to develop colitis-associated CRC compared to the general population [31,32]. Recently, a study on 44,278 individuals showed an association between a higher dietary inflammatory index, which is developed to evaluate the inflammatory potential of an individual’s diet, and an increased prevalence of colorectal adenomas [33]. The consumption of non-steroid anti-inflammatory drugs, such as aspirin, was shown to reduce the occurrence of CRC and decrease tumor growth in various animal models of CRC [34]. Moreover, the susceptibility to develop colonic tumors in animal models of CRC, such as *APC^Min/+^* mice (which carry a germline mutation in *Apc* gene) and AOM-treated mice, is enhanced following treatment with the inflammatory agent dextran sodium sulfate (DSS) [35,36]. It is well known that chronic inflammation induces dysplasia via the induction of DNA modifications in IECs, such as nitration, oxidation, methylation and deamination reactions, which can contribute to the initiation or progression of CRC [37]. During inflammation, the recruitment of innate immune cells such as macrophages, neutrophils and dendritic cells and adaptive immune cells such as T and B cells, leads to the secretion of oxygen/nitrogen reactive species, which are highly genotoxic [38], pro-inflammatory cytokines such as interleukin (IL)-6, IL-8, IL-1β and tumor necrosis factor-α (TNF-α), as well as growth factors [39]. The production of these mediators is mediated by several major signaling pathways such as nuclear factor-kappa B (NF-κB), signal transducer and activator of transcription 3 (STAT3), PI3K/AKT, cyclo-oxygenase-2 (COX-2)/prostaglandin E2 (PGE2), which are implied in many processes including proliferation, angiogenesis, invasion, metastasis and recruitment of inflammatory mediators [39]. This inflammatory environment has a lot of similarities with the tumor microenvironment, suggesting the implication of the same mediators in chronic intestinal inflammation and colorectal carcinogenesis [39]. Indeed, many inflammatory mediators have been found positively associated with the prevalence of colorectal adenomas [40,41,42]. For example, IL-6 levels are higher in the serum of CRC patients compared to healthy controls [43]. In vitro, IL-6 was shown to stimulate the invasiveness of human colorectal carcinoma cells [44]. Using a mouse model of AOM-DSS-induced colitis-associated CRC, IL-6 was also shown to be a strong promoter of colonic tumor growth [45]. Mice deficient for the anti-inflammatory cytokine IL-10 (*il10^−/−^* mice), which develop spontaneously chronic colitis [46], have increased carcinogenesis with higher grade and invasiveness when being treated with AOM compared to wild type mice [47]. In addition, IL-10 deficiency leads to increased colon tumor number in *APC^Min/+^* mouse model of CRC [48]. Interestingly, under germ-free condition *il10^−/−^* mice develop reduced colitis, and this is associated with reduced AOM-induced CRC development [47]. Moreover, the intestinal microbiota composition is different in AOM-treated *il10^−/−^* mice compared to AOM-treated wild type mice [49]. 

The implication of toll-like receptors (TLRs) and nucleotide-binding oligomerization domain (NOD)-like receptors (NLRs), which are innate immune sensors that function to maintain gut homeostasis by inducing an appropriate inflammatory response against pathogenic exposures, in inflammation-associated colorectal carcinogenesis has been largely investigated. Ten TLRs have been identified in humans, and several single nucleotide polymorphisms (SNPs) within the *tlr* genes have been associated with altered susceptibility to infectious, allergic, and inflammatory diseases as well as cancers [50]. A correlation between SNPs in *tlr3*, *tlr5* and *tlr9* genes and CRC has been found [51,52]. A dual role for TLRs in CRC has been proposed as they may promote cancer cell survival and progression or induce tumor cell death depending on the context [53]. For example, TLR5 and TLR9 exhibit anti-tumoral properties by activating immune cells and having a direct cytotoxicity effects on tumor cells [53]. Moreover, TLR8 activation was shown to inhibit regulatory T cells, thus promoting anti-tumor immunity [54]. Using a mouse xenograft model of human colon cancer, a study showed that deficiency of TLR5 is associated with increased tumor volume accompanied with a deregulation of tumor immune response [55]. In addition, TLR9 exhibits also anti-tumoral activity in a xenograft model of colon cancer [56]. Finally, TLR2 deficiency leads to increased tumor development with higher pro-inflammatory mediators’ level in an AOM-DSS mouse model of inflammation-induced CRC [57]. In contrast, TLRs have the capacity to activate the NF-κB signaling pathway, and this is one of their major tumor-promoting effects [53]. TLR activation stimulates several immune mediators, such as IL-1β, TNF-α and IL-6, which are implied in cell survival, immune response and inflammation [53]. In vitro, TLR4 was found to enhance immunosuppression by inhibiting T cell proliferation [58]. Using xenograft mouse model of CRC, the blockade of TLR4 was found to improve the survival of tumor-bearing mice [58], and this was confirmed in the AOM-DSS mouse model, where TLR4 was shown to recruit and activate COX-2-expressing macrophages and increase the number and size of dysplastic lesions per colon [59]. Moreover, deficiency of the TLR adaptor molecule myeloid differentiation primary response gene 88 (MyD88) in *APC^Min/+^* mice leads to a decrease in the number of colonic and ileal polyps [60]. 

In 2004, Kurzawski and colleagues fist reported an association between a SNP in *nod2* gene and an enhanced risk to develop CRC [61]. It was later shown that NOD2 deficiency increases the susceptibility of mice to chemically induced colitis and colitis-associated carcinogenesis, and this is due to changes in the composition of gut bacterial communities and enhanced IL-6 production [62]. Deficiency of NOD1 leads to increased colorectal tumor number in *APC^Min/+^* mice and AOM-DSS-treated mice. Treatment with antibiotics suppresses intestinal tumor formation in NOD1-deficient mice compared to untreated mice [62]. Moreover, following AOM-DSS treatment, NOD1-deficient mice exhibit impaired interferon gamma (IFN-γ) production and therefore increased inflammation-associated tumorigenesis compared to wild type mice [63].

These data suggest a close link between inflammation and microbiota modulation during colorectal tumorigenesis.

## 3. Intestinal Microbiota and Gut Homeostasis 

The intestinal microbiota is the complex community of all microorganisms in the gut, including not only bacteria but also fungi, viruses, archaea and protozoans. It has been estimated that over 1000 bacterial species inhabit the human intestinal tract [64]. In healthy individuals, the microbiota is mainly composed of two principal strictly anaerobic phyla: the *Firmicutes* and the *Bacteroidetes*. Despite the stability of these groups in the gut, their proportions and the associated species are highly variable over time and between individuals [65]. In a single individual, there is also a spatial variability in the composition and the amount of microbiota. Indeed, it has been observed an increase in the number of bacteria beginning at 10–10^3^ bacteria per gram of stomach and duodenal contents, increasing to 10^4^–10^7^ bacteria per gram in the small intestine, and rising to 10^11^–10^12^ bacteria per gram in the large intestine [66]. The gut microbiota has a symbiotic relationship with the host and is involved in metabolic, immunological and protective functions in a healthy individual. This part lists the main functions of the healthy intestinal microbiota.

### 3.1. Nutrient Metabolism

The gut microbiota has a major role in metabolism by providing important metabolites for its host. The key bacterial fermentation products following the fermentation of dietary carbohydrates are SCFAs and gases. SCFAs, such as butyrate, propionate, and acetate, are the main end products synthesized from the fermentation of non-digestible carbohydrates by the two main fermenters: *Bacteroidetes* which transform simple sugars from carbohydrates into organic acids such as SCFAs and hydrogen, and *Clostridium* with butyrate-producing bacteria that transform organic acids into additional SCFAs. The beneficial roles of SCFAs for the host have attracted many researchers, such as their role in energy homeostasis as they are the principal source of energy for colonocytes [67], their anti-inflammatory and anti-carcinogenic effects, and their capacity to reinforce the intestinal barrier function and to decrease the oxidative stress [9]. The gut microbiota plays also a role in gas metabolism. The majority of gas generated by bacteria comprises hydrogen, carbon dioxide, and methane, all odorless gases. Gas production by the colonic microbiota can exert clinical consequences for the host. For example, the utilization of hydrogen to reduce sulfate generates hydrogen sulfide, which is highly toxic to colonocytes and can have pathological consequences. There is also an association between the presence of methane in the colon and CRC, although this could be a consequence rather than causal of the disease [68].

The gut microbiota has also an impact on lipid metabolism as the microbiota can enhance the lipoprotein lipase activity in adipocytes [69]. The lipids can be derived from the intestine itself, from the desquamation of the epithelial cells and from the bacteria [70]. Only 5% of bile acids, transformation products from cholesterol, reach the colon to be metabolized by bacteria into secondary bile acids. *Bacteroides intestinalis*, for example, has the ability to deconjugate and dehydrate the primary bile acids to convert them into secondary bile acids in the colon [71]. Several primary bile acids such as cholic acid are converted into desoxycholic acid and lithocholic acid and may have carcinogenic effects [72]. The gut microbiota has also a role in protein metabolism. Indeed, a lot of bacteria have protease activity and can hydrolyze proteins into small peptides [73]. These peptides can be then metabolized by several bacteria into amino acids which can serve as a source of energy or nitrogen by other bacteria [73]. The gut microbiota can also synthesize certain vitamins, notably vitamins K and B, which are not only important for bacterial metabolism, but also have a physiological significance to the host [74]. For example, people treated with a broad-spectrum antibiotic showed a significant decrease in plasma prothrombin levels [75]. Germ-free but not the conventional animals fed a diet without vitamin K supplement have low prothrombin levels and develop hemorrhages [76].

### 3.2. Intestinal Barrier Maintenance

The principal functions of the intestinal epithelium are to form a barrier and protect the gut from the external environment, to regulate the absorption of nutrients, electrolytes and water from the lumen and to maintain the homeostasis between the environment and the host. In order to maintain a high protection, the intestinal epithelium is composed of two main elements: the mucus layer and the tight junctions. The gut microbiota has an impact on both of them. Indeed, it has been shown that the mucus layer is not well developed in germ-free mice [77]. Moreover, the SCFAs produced by the gut microbiota and more specifically butyrate can act as a guardian of the intestinal barrier by decreasing the permeability through increased expression of the tight junction proteins claudin-1 and zonula occludens-1 [78]. SCFAs such as butyrate have also an impact on intestinal mucus production by enhancing expression of mucins [79]. Germ-free animals show impaired intestinal barrier due to decreased tight junction protein expression and low expression of mucus proteins, and therefore a high susceptibility to DSS-induced colitis [80]. These studies show a major role of the microbiota in protecting the gut integrity.

### 3.3. Modulation of Immune System

The gut microbiota contributes to the maturation and modulation of both mucosal and systemic immune systems via innate immune components not much specific such as the pattern recognition receptors (PRRs) expressed on the different cell types in the mucosa (enterocytes, polynuclear cells, mast cells, macrophages and dendritic cells), and adaptive immune components which are highly specific receptors expressed on the surface of T cells and B cells. Recruitment and activation of all of these cells are highly dependent on signals from the microbiota and are tightly regulated. 

#### 3.3.1. Intestinal Innate Immune Cells

Among the innate immune cells, macrophages are the most abundant. In the intestine, macrophages have a phagocytic activity by expressing the phagocytic receptor TREMC2 (triggering receptor expressed on myeloid cells 2), and therefore the ability to get rid of invasive bacteria [81]. Macrophages are also producers of the anti-inflammatory cytokine IL-10 which contributes to the maintenance of intestinal homeostasis [81]. Neutrophils and eosinophils play also a role in innate immunity by respectively secreting the pro-inflammatory mediators such as IL-22 and stimulating the adaptive immune responses via the production of immunoglobulin-A (IgA) [82,83]. Innate lymphoid cells are activated in response to cytokines produced by dendritic cells or by the epithelium. Among these cells, the type 3 innate lymphoid cells (ILC3) expressing the nuclear factor retinoid acid-related orphan receptor γ, which are activated by IL-1β, IL-6 and IL-23, are producers of effector cytokines such as IL-17 and/or IL-22, and require the presence of commensal bacteria for their development [84]. When being activated, ILC3 have also the ability to induce the production of mucus and antimicrobial peptides (AMPs) by the epithelium. Moreover, ILC3 have a direct impact on adaptive immune response through the production of granulocyte macrophage colony-stimulating factor (GM-CSF). GM-CSF production, as a consequence of the detection of commensal bacteria and the production of IL-1β by macrophages, leads to the generation of regulatory T cells [85]. ILC3 are also found to express major histocompatibility complex molecules, process and present antigens, and interact with CD4^+^ T cells leading to the regulation of adaptive immune responses to commensal bacteria [86]. Finally, dendritic cells are key regulators of adaptive immune responses by recruiting and activating naïve T cells by inducing T cell receptors [87]. One subpopulation of dendritic cells is predominant in Peyer’s patches, key site of microbiota-induced immune responses, and could promote regulatory T cell production, while the other subpopulation seems to have pro-inflammatory properties by promoting T cell repertory [87]. 

#### 3.3.2. Intestinal Adaptive Immune Cells

Peyer’s patches and isolated lymphoid follicles are the major sites for adaptive immune responses. These two sites are enriched in microfold cells (M cells), which allow the translocation of bacteria that can be captured by dendritic cells and presented to naïve T cells, leading to the activation of B cells and therefore the secretion of IgA [88]. Compared to conventional animals, germ-free animals have reduced number and unachieved development of Peyer’s patches. Indeed, Peyer’s patches from germ-free mice exhibit fewer M cells and T lymphocytes [89]. Germ-free mice have also decreased IgA-producing plasma cells and reduction of T cells in the *lamina propria* [90]. Bacterial colonization induces the production of IL-17 by the T helper 17 (Th17) cells, which is important to control intestinal bacteria. Indeed, IL-17 stimulates the production of AMPs by the epithelium, the recruitment of neutrophils, and also promote IgA secretion [91,92]. Immune responses vary according to bacterial densities and are dependent of the microbial community. The most implied bacteria in the modulation of both innate and adaptive immune systems are the segmented filamentous bacteria (SFB) [93]. The SFB, related to *Clostridium*, adhere to the epithelial surface and to the Peyer’s patches in order to get nutrients [94]. This contact between the SFB and the epithelium is also beneficial for the host by stimulating the immune system. Indeed, the SFB stimulate innate immune responses and promote the development of lymphoid tissues such as Peyer’s patches and the isolated lymphoid follicles. SFB also induce IgA secretion and activate pro-inflammatory T cells as well as regulatory T cells [91,95].

Besides the role in shaping the intestinal immune system, the gut microbiota has also indirect effects on the periphery. While the mechanisms are still poorly described, theories have emerged suggesting that the gut microbiota might have peripheral effects by the diffusion of soluble factors derived from bacteria and their metabolites [96,97]. Indeed, Burgess and colleagues showed that transfer of bone marrow-derived macrophages from mice carrying SFB to mice deficient in SFB is sufficient to protect SFB-deficient mice from infection with *Entamoeba histolytica*, responsible of diarrhea [98]. Furthermore, it was shown that the gut microbiota can protect against enteric infection via extra-intestinal mediators [98].

### 3.4. Protection against Pathogens

Studies have shown a crucial role of gut microbiota in protection against gut colonization by pathogens. It has been shown that antibiotic-treated mice have increased susceptibility to infection with enteric pathogens compared to untreated mice [99,100]. The mechanisms by which the gut microbiota inhibits gut colonization by pathogens involve competition for adhesion receptors and for nutrients, stabilization of the mucosal barrier and production of anti-microbial substances [101]. 

Commensal microbiota and bacterial pathogens require the same niche to colonize the intestine. Commensal bacteria are able to produce bacteriocins and toxins that inhibit specifically the members of the same species. For example, bacteriocin produced by several commensal *Escherichia coli* strains isolated from human and different animals inhibits the growth of the pathogenic enterohemorrhagic *Escherichia coli* (EHEC) [102]. Moreover, commensal bacteria have the ability to influence the pH of the gut in order to prevent the colonization of pathogens. For example, *Bifidobacterium* protect mice against death induced by EHEC serotype O157:H7 through acidification of the environment via the production of acetate [103]. Similarly, the SCFAs produced by some commensal bacteria can have toxic effects for some pathogens such as *Salmonella* by modifying the environment pH [67]. Moreover, SCFAs, especially butyrate, have been shown to inhibit the virulence of *Salmonella* by decreasing the *Salmonella* pathogenicity island 1 gene expression, thereby limiting the invasion of epithelial cells by this pathogen [104]. Another strategy used by commensal bacteria to inhibit the colonization by pathogens is the competition for nutrients, leading to starvation of pathogenic bacteria. Indeed, a study showed that co-culture with high proline-consuming commensal *E. coli* decreases the growth of EHEC serotype O157:H7 [105]. Moreover, the modulation of the microenvironment in the gut, such as oxygen concentration, by commensal bacteria can lead to incomplete virulence gene expression in pathogens such as *Shigella flexneri* [106]. 

Another defense strategy from the commensals against pathogens is the activation of host innate immunity via the pathogen-associated molecular patterns (PAMPs) or microbe-associated molecular patterns (MAMPs) which include microbial components such as lipopolysaccharides, lipid A, flagella, bacterial DNA and RNA [107]. Those PAMPs/MAMPs are recognized by the PRRs such as the TLRs, the C-type lectin receptors (CLRs) and the NLRs of eukaryotic cells. The interactions between PRRs and PAMPs/MAMPs lead to the activation of several pathways guaranteeing the intestinal homeostasis such as those implied in the mucosal barrier function or in the synthesis of AMPs by Paneth cells such as C-type lectins, prodefensins and cathelicidins [108]. *MyD88*^−/−^ mice have impaired production of AMPs by Paneth cells in the small intestine, leading to enhanced colonization by commensal bacteria in the mesenteric lymph node and also an increased dissemination of the pathogenic bacterium *Salmonella* into the spleen [109]. Mice deficient for the intracellular sensor of small bacterial peptides NOD2 show an impaired production of the α-defensins, called cryptdins in mice, which might lead to a higher susceptibility to infection by pathogens [110]. Using mice deficient for MyD88 specifically in IECs (MyD88^∆IEC^ mice), a study showed that the loss of MyD88 results in an increased number of mucosa-associated bacteria, impaired mucus-associated antimicrobial activity, increased bacterial translocation, decreased mucin-2 expression, and decreased expression of epithelial IgA transporter, leading to an enhanced susceptibility of mice to colitis [111]. In addition, Frantz et al. also noted a significant difference in the gut microbiota composition in MyD88^∆IEC^ mice, with a decrease in the abundance of *Bacteroides* and an increase in a large proportion of species belonging to *Proteobacteria*, compared to the control MyD88^flox/flox^ littermates [111]. Among the commensal bacteria, some species of *Bacteroides* and *Lactobacillus* are the more frequently implied in the production of AMPs [66]. Mono-colonization of germ-free mice deficient in T cells and IgA secretion (RagT mice) with either the gram-negative *Bacteroides thetaiotaomicron* or the gram-positive *Lactobacillus innocua* results in a significant increase in mRNA expression of RegIII-gamma, which is a secreted C-type lectin, part of the AMP families, triggered by enhanced mucosal contact between bacteria and epithelial cells [112]. Using germ-free mice mono-colonized with SFB, which are specific members of the commensal microbiota, Ivanov and colleagues showed that SFB are able to induce Th17 cells in the *lamina propria* and production of the Th17 cell effector cytokines IL-22 and IL-17 [91]. These are accompanied by a decrease in the invasion of the pathogen *Citrobacter rodentium* in the colonic tissue compared to germ-free mice that are not mono-colonized with SFB and therefore lack Th17 cells [91]. However, some pathogens have developed strategies to use commensal bacteria for their own good. For example, *Clostridium difficile* use bile salt, a by-product derived from commensal bacteria, in order to stimulate the germination of spores [113]. 

## 4. Intestinal Microbiota and CRC

In 2012, among the 14 million new cancer cases, 2.2 million cases were attributed to infectious agents [114]. A review summarizing all the epidemiologic and pathologic studies since 2000 showed that the proportion of cancer cases attributed to infectious agents is up to 20%. This varies greatly from 5% in highly developed countries to more than 50% in Sub-Saharan African countries where 90% of the cancer cases attributed to infection were caused by *Helicobacter pylori* (770,000 cases), human papillomavirus (640,000 cases), hepatitis B virus (420,000 cases), hepatitis C virus (170,000 cases) and *Epstein-Barr* virus (120,000 cases) [114,115]. Since 99% of the microbial mass is located in the intestinal tract, the gut microbiota has the greatest impact on human health and is the most studied microbiota. Several studies have shown a link between a modification of the gut microbiota and CRC. In 1995, a study reported 15 bacterial species associated with a higher risk to develop CRC, including two *Bacteroides* species (*Bacteroides vulgatus* and *Bacteroides stercoris*), two *Bifidobacterium* species (*Bifidobacterium longum* and *Bifidobacterium angulatum*), five *Eubacterium* species (*Eubacterium rectale* 1 and 2, *Eubacterium eligens* 1 and 2, *Eubacterium cylindroides*), three *Ruminococcus* species (*Ruminococcus torques*, *Ruminococcus albus* and *Ruminococcus gnavus*), *Streptococcus hansenii*, *Fusobacterium prausnitzii* and *Peptostreptococcus productus* 1 [116]. The authors also reported five bacterial species associated with a lower risk of CRC development including some *Eubacterium* species, *Lactobacillus* S06, *Peptostreptococcus* DZ2 and *Fusobacterium* AB [116]. By analyzing the microbiota composition of different intestinal compartments from 46 patients with CRC and 56 healthy volunteers, Chen and colleagues showed that the mucosa-associated bacterial composition was significantly different in CRC patients compared to healthy subjects [5]. Indeed, *Fusobacterium*, *Porphyromonas*, *Peptostreptococcus*, *Gemella*, *Mogibacterium* and *Klebsiella* are enriched in CRC patients, whereas *Feacalibacterium*, *Blautia*, *Lachnospira*, *Bifidobacterium* and *Anaerostipes* are reduced [5]. Moreover, the authors showed that the microbiota of cancerous tissues exhibited lower diversity compared to that of the non-cancerous normal tissues [5]. More recently, Goa et al. showed that the predominant phylum in CRC patients is the *Firmicutes*, whereas it is the *Proteobacteria* in healthy individuals. In addition, a relatively higher abundance of *Lactococcus* and *Fusobacterium* and lower abundance of *Pseudomonas* and *Escherichia-Shigella* was observed in cancerous tissues compared to adjacent non-cancerous tissues [7]. Recent pyrosequencing data of CRC-associated gut microbiota revealed, in particular, over-representation of some bacteria such as *Bacteroides/Prevotella*, *Faecalibacterium* and *Fusobacterium* [117]. However, these modifications vary depending on the analysis techniques and the sample localization. Indeed, Sobhani and colleagues showed that *Bacteroides* are over-represented in CRC patients’ tissues (tumoral tissues and associated normal mucosa) compared to normal tissues from control subjects. In the stool samples, the authors showed a significant increase of *Bacteroides/Prevotella* in CRC samples compared to healthy subjects’ samples [118]. When analyzing CRC at an earlier stage, studies have shown an increase of *Proteobacteria* and *Fusobacteria* and a decrease of *Bacteroides* in normal mucosa from CRC patients compared to that from control subjects [119,120]. At species levels, *Bacteroides fragilis*, *Escherichia coli*, *Streptococcs bovis/gallolyticus*, *Enteroccocus faecalis* and *Fusobacterium nucleatum* are increased in the fecal samples from CRC patients, while *Bacteroides vulgatus* and *Faecalibacterium prausnitzii* are decreased when compared to fecal samples from healthy volunteers [117,121]. More recently, Viljoen and colleagues reported a significant increase in *Fusobacterium* in tumor samples compared to non-tumoral adjacent mucosa, and this is associated with late stages of CRC [122]. The alterations in intestinal microbiota composition have also been found in animal models of CRC. Indeed, in 2013, using the AOM-DSS mouse model of colitis-induced CRC, Zackular and colleagues showed a shift in fecal microbiota composition with a significant decrease in the diversity following the first round of DSS treatment [123]. Right after the first round of DSS treatment, *Bacteroides* was found increased, while *Prevotella* was found decreased [123]. However, following the third round of DSS treatment, a significant decrease in *Bacteroides* and *Porphyromonadaceae* was found, which has also been observed in IBD patients [123,124]. The authors proposed that these species could have a protective role as the anti-inflammatory mediators in the gut. When they conventionalized germ-free mice with either the healthy microbiota of untreated mice or the microbiota of tumor-bearing AOM-DSS-treated mice, those conventionalized with tumor-bearing mice-associated microbiota exhibit more tumors and decreased gut microbiota diversity compared to those conventionalized with the healthy microbiota [123]. Analyses of the diversity and richness of the intestinal lumen microbiota were also performed via the analysis of the feces in an animal model of CRC induced by the carcinogenic agent 1,2-dimethylhydazine [125]. The results showed an increase in *Bacteroides* and *Proteobacteria* in the lumen of CRC rats compared to healthy rats. A reduction of butyrate-producing bacteria such as *Roseburia* and *Eubacterium* in the gut microbiota of CRC rats was also observed [125]. Recently, it was shown that germ-free *APC^Min/+^*/*il10*^−/−^ mice exibit almost no tumor compared to conventionalized *APC^Min/+^/il10*^−/−^ mice, indicating the primordial role of the gut microbiota in inflammation-induced CRC [48]. 

Theories have been made regarding the role of the gut microbiota in CRC initiation or progression. Tjalsma and colleagues proposed a “driver-passenger” bacterial model, in which the intestinal mucosa of CRC patients could be colonized by one or several microbes called “driver” because of their pro-carcinogenic properties such as production of DNA-damaging compounds, induction of cellular proliferation, causing permeabilization of intestinal barrier and induction of chronic inflammation, leading to initiation of CRC. *Enterococcus faecalis*, some *Escherichia coli* strains, *Bacteroides fragilis*, *Shigella*, *Salmonella* and *Citrobacter* have been described among the “driver” bacteria [4]. The “driver” bacteria are associated with the early stages of CRC and are not found in cancerous tissue as the disease progresses, which may explain the heterogeneity of the results reported by CRC-associated microbiota studies. Via their pro-carcinogenic effects, the “driver” bacteria can influence the tumoral microenvironment and promote the emergence of “passenger” bacteria, which are better suited to the new environment. *Fusobacterium nucleatum*, *Streptococcus bovis/gallolyticus* and with less evidence *Clostridium septicum* have been considered as candidate “passenger” bacteria [4]. Primarily linked to gastric cancer, studies have also started to investigate the association between *Helicobacter pylori* and CRC [126].

## 5. Possible Mechanisms of Action of the Intestinal Microbiota in Colorectal Carcinogenesis

### 5.1. Enterococcus faecalis

*E. faecalis* is a gram-positive facultative anaerobic commensal bacterium and mostly appears harmless to humans. However, studies have started to associate *E. faecalis* to CRC because it has been found to be enriched in fecal samples from CRC patients compared to healthy individuals [127], and also in tumors as well as in the adjacent tissues of CRC patients compared to mucosa from healthy individuals [128]. Recently, a study reported the case of an 86-year-old Caucasian male with *E. faecalis* bacteremia, who presented gastrointestinal bleeding secondary identified to be colorectal adenocarcinoma by colonoscopy [129]. In *il10*^−/−^ mice, *E. faecalis* was shown to be able to promote and perpetuate colitis, to induce dysplasia and rectal carcinoma [130]. It was also shown that upon infection with colitogenic *E. faecalis*, IECs from wild type mice express the immunosuppressive cytokine TGF-β, thus activating Smad signaling [131]. This was associated with a loss of TLR2 protein expression and inhibition of NF-κB-dependent pro-inflammatory gene expression. In contrast, *il10*^−/−^ mice fail to inhibit TLR2-mediated expression of pro-inflammatory genes in IECs upon colonization with *E. faecalis* [131]. In addition to its ability to induce chronic inflammation, *E. faecalis* was shown to produce extracellular superoxide and hydrogen peroxide [132]. In vitro, this production of extracellular free radical was shown to induce DNA damage [133]. When being administered to rats, *E. faecalis* is also able to induce DNA damage in luminal colonic cells [133]. Since reactive oxygen species (ROS) are able to induce chromosomal instability [134], which could be associated with CRC occurrence, a study investigated whether *E. faecalis* could promote CIN [135]. Using mammalian cells, the authors showed that *E. faecalis* is able to induce CIN, and this is due to the production of superoxide but not hydrogen peroxide, and this seems to involve COX-2 whose expression is enhanced after 2 h of infection. The authors admitted that extracellular superoxide-producing *E. faecalis* infection leads to enhanced COX-2 expression in macrophages and promotes CIN in epithelial cells [135]. More recently, Wang and colleagues showed that *E. faecalis* is able to polarize colon macrophages to a M1 phenotype. *E. faecalis*-polarized macrophages were shown to induce aneuploidy and chromosomal instability in primary colon epithelial cells which are commonly found in cancers [136]. In addition, primary murine colon epithelial cells when being repetitively exposed to *E. faecalis*-infected macrophages are transformed with strong expression of stem/progenitor cell markers. In immunodeficient mice, eight of 25 transformed clones grow as poorly differentiated carcinomas with three tumors invading skin and/or muscle [136]. These findings could explain the mechanisms by which *E. faecalis* exert son impact on colorectal carcinogenesis. 

### 5.2. Bacteroides fragilis

The strict anaerobe *B. fragilis* is a common human symbiont that colonizes the entire length of the colon and represents only a small proportion of the gut microbiota. There are two subtypes of *B. fragilis*, the nontoxigenic *B. fragilis* (NTBF) and the enterotoxigenic *B. fragilis* (ETBF). The latter, which has been associated to diarrhea in humans [137], exhibits a pathogenic island, called the *B. fragilis* pathogenicity island (BfPAI), that allows them to produce an enterotoxin called “fragilysin” or BFT encoded by the *bft* gene [138]. Several studies have linked *B. fragilis* with CRC as it has been found enriched in stools from CRC patients compared to healthy individuals [117,118]. Using stool samples from 73 CRC patients and 59 healthy subjects, the *bft* gene has been found in 38% of the CRC patients’ samples compared to 12% in the healthy group [139]. ETBF is associated with late-stage CRC as 100% of the late-stage tumors are *bft*-positive compared to 72% of the early-stage tumors [140]. However, Purcell and colleagues showed that *B. fragilis* is associated with early-stage carcinogenic lesions [141]. In vitro studies have highlighted the proteolytic activity of fragilysin, which is responsible for the degradation of tight junction proteins such as zonula occludens-1 [142] and therefore leads to a dysfunction of the intestinal epithelial barrier with enhanced epithelial permeability and damaged intestinal crypts and colonocytes [143,144]. In 2003, Wu and colleagues showed that EBFT is able to degrade the cellular adhesion molecule E-cadherin in HT29 cells, triggering the translocation of β-catenin into the nucleus and the transcription of the oncogene c-myc, leading to enhanced and persistent cellular proliferation that could positively influence CRC development [145]. In *APC^Min/+^* mice, ETBF colonization leads to an increase in colonic thickness, inflammation and visible colonic tumors, which were not observed with NTBF infection [146]. ETBF mediates its effects via the activation of STAT3 in colonic epithelial cells and therefore induces the pro-carcinogenic Th17 inflammatory response with subsequent secretion of the pro-inflammatory cytokine IL-17. When blocking the IL-17 secretion with IL-17 neutralizing antibodies, EBTF-induced colon tumors are significantly reduced without affecting STAT3 activation, showing the preponderant role of EBFT-induced inflammation in the promotion of colon carcinogenesis [146].

### 5.3. Fusobacterium nucleatum

*F. nucleatum* is a gram-negative strictly anaerobic oral commensal and periodontal pathogen associated with diverse diseases [147]. *F. nucleatum* has recently been associated with CRC as its prevalence is enhanced in mucosa from patients with CRC compared to control subjects [120] and is found in higher proportion in CRC tumors compared to adjacent normal tissues [148,149]. *F. nucleatum* administration leads to increased tumor size and number, ascites, diarrhea, gut dilatation, splenomegaly and also shorter survival in *APC^Min/+^* mice. The tumors from *APC^Min/+^* mice infected with *F. nucleatum* exhibit high levels of proliferating cell nuclear antigen compared with uninfected *APC^Min/+^* mice, indicating the positive impact of *F. nucleatum* on cell proliferation [150]. *F. nucleatum* infection also leads to activation of the immune response with increased levels of inflammatory mediators in the serum of infected *APC^Min/+^* mice compared to uninfected group [150]. In addition, *F. nucleatum* infection induces expression of miRNA 21, which is considered as “oncomiR” because of its oncogenic properties [150,151]. Gene expression microarray analysis showed activation of the TLR4/MYD88/NF-κB pathway in colon cancer cells upon infection with *F. nucleatum*, and in vitro experiments confirmed that *F. nucleatum* regulates miRNA 21 expression via the TLR4/MYD88/NF-κB pathway [150]. Using *APC^Min/+^* mice, *F. nucleatum* was shown to be able to increase tumor development, without inducing colitis, accompanied with increased infiltration of myeloid cells into the tumors [152]. Assessment of the tumor immune microenvironment showed that compared to the uninfected group, *APC^Min/+^* mice infected with *F. nucleatum* exhibit enhanced proportion of myeloid-derived suppressor cells, which are tumor permissive myeloid cells, increased tumor-associated neutrophils, which are known to play a role in tumor progression, an enrichment of tumor-associated macrophages, which are also known as promoters of carcinogenesis, and an increase in dendritic cells, which have a role in anti-tumor immunity [152]. This pathogen is also able to invade epithelial cells via its virulence factor FadA by modulating the E-cadherin signaling pathway, leading to the activation of several transcription factors such as T-cell factor (TCF), β-catenin, NF-κB, c-myc and cyclin D1 and subsequently enhanced proliferation of colon cancer cells [153]. Using xenograft model, it was shown that FadA is able to enhance tumor growth and induce the release of pro-inflammatory cytokines, and this is mediated by E-cadherin [153]. These data suggest that *F. nucleatum* may not only impact the tumor microenvironment but has also a more direct impact on the tumor [153]. 

### 5.4. Streptococcus bovis/gallolyticus 

The association between *S. bovis* and CRC was first been made in 1951 [154]. In 1977, *S. bovis* was isolated from fecal samples from 35 of 63 CRC patients compared to 11 of 105 control individuals with no apparent gastrointestinal diseases, showing the high prevalence of this bacterium in CRC patients [155]. Since then, a lot of studies have confirmed the link between *S. bovis/gallolyticus* and CRC [156,157]. Studies have shown that *S. bovis* is implied in various cellular and molecular modifications that could be linked to the development of CRC. An in vitro study showed that infection of colon cancer epithelial cells with *S. bovis* leads to the increased expression of pro-inflammatory mediators such as IL-8, COX-2 and the release of PGE2 [158]. Experiments using AOM-treated rats confirmed the release of pro-inflammatory mediators following the infection with *S. bovis*, which leads to increased number of aberrant crypts. Three of six AOM-treated rats developed polyps following *S. bovis* infection, whereas no polyp was found in uninfected AOM-treated rats [158]. Another study using AOM-treated rats highlighted the ability of *S. bovis* to promote colorectal carcinogenesis by enhancing proliferation markers leading to increased number of hyper-proliferative crypts [159]. Using human samples (feces, mucosa, tumorous and non-tumorous colorectal tissues), Abdulamir and colleagues showed an enrichment of this bacterium in fecal and mucosal samples of CRC patients compared to control subjects without gastrointestinal lesions, reinforcing the link between *S. bovis* and CRC [160]. Moreover, *S. bovis* is found with higher proportion in tumoral tissues compared to the non-tumoral one [160]. In addition, the authors showed significant higher mRNA expression levels of pro-inflammatory mediators (IL-1β, COX-2, and IL-8) in *S. bovis*-infected tissues compared to uninfected tissues, but also higher in tumorous tissues compared to the non-tumorous one, highlighting a possible role of *S. bovis* in inflammation-induced CRC [160]. 

### 5.5. Clostridium septicum

*Clostridium septicum* is an aerotolerant, gram-positive, pore-forming bacillus not usually present in the normal intestinal flora of humans. *C. septicum* produces a virulence factor, α-toxin, which is both lethal and hemolytic [161]. Only rare bacteremia are attributed to *C. septicum* (1%) with high rate mortality (60%) [162]. The association of *C. septicum* with CRC has been suggested [163,164,165]. This association could be explained by the fact that the germination of *C. septicus* spore could be favored by the hypoxic and acidic tumor environment [163]. The exact mechanisms underlying the contribution of this bacterium in colorectal carcinogenesis are still poorly known. Recently, a study showed the ability of α-toxin-producing *C. septicum* to induce activation of mitogen-activated protein kinase (MAPK) signaling, which has been shown to be deregulated in various diseases including cancers. This activation is associated with a release of the pro-inflammatory cytokine TNF-α [166], which could lead to a pro-inflammatory environment propitious for cancer development. Despite these data, no direct link between *C. septicum* and CRC has been defined. 

### 5.6. Helicobacter pylori

*H. pylori* is a gram-negative bacterium that colonizes specifically the gastric epithelium of slightly more than 50% of the population. Although most of the infected population remain asymptomatic, *H. pylori* is known to induce chronic inflammation and is a risk factor for the occurrence of gastric ulcer, mucosa-associated lymphoid tissue (MALT) lymphomas and gastric adenocarcinomas [167]. Even if the colonization of *H. pylori* is located in the stomach, it has been demonstrated that its toxicity can be extra-gastric [168]. The association between *H. pylori* infection and CRC is still controversial with studies showing a close link with a higher prevalence of *H. pylori* infection in patients with colonic adenomas and carcinomas [169,170,171,172], while others do not [173,174,175]. Recent studies released in 2017 have suggested indeed a significant association between *H. pylori* infection and an increase in CRC occurrence [176,177,178]. Yan and colleagues showed a positive association between *H. pylori* and CRC only when *H. pylori* is associated with intestinal metaplasia [178]. Analyzing 1245 colorectal adenomas and 3221 control subjects without polyp, Nam and colleagues showed that the overall rate of positive *H. pylori* infection is increased in adenoma cases compared to polyp-free control cases, and that the positive association of *H. pylori* infection with colorectal adenomas is more prominent in advanced adenomas and multiple adenomas [177]. Despite this controversy, some studies have tried to clarify the mechanism underlying the potential association between this pathogen and CRC with some hypotheses including release of toxin or hormone, intestinal microbiota fluctuation and chronic inflammation. Indeed, increased levels of gastrin, an important hormone of the digestive system that assists gastric acid secretion, in *H. pylori*-infected patients was shown [179]. The *H. pylori*-induced over-production of gastrin is associated with enhanced COX-2 expression and reduced apoptosis due to increased expression of the anti-apoptotic protein BCL2 over the pro-apoptotic protein BAX [179]. In vivo, supplementation of gastrin leads to increased proliferative index in the colon, expansion of the proliferative zone in the intestinal crypt, increased thickness of the colonic mucosa and hyperplasia of goblet cells, which may increase the risk to develop CRC [180]. The perturbation in acid production generated by the over-production of gastrin might be linked to a gastric barrier perturbation, which can lead to fluctuation in gut microbiota [181]. Studies have shown that this perturbation can facilitate the colonization and growth of CRC-associated bacteria such as *B. fragilis* and *E. feacalis* [181]. Another theory is that the production of ROS and reactive nitrogen species (RNS) by *H. pylori* can lead to DNA damage, which could favor colorectal carcinogenesis [182]. Furthermore, different strains of *H. pylori* have different impacts on patients. Indeed, the strains that exhibit the virulence factor CagA are more harmful than those without this factor, and patients carrying these strains have an increased risk to develop gastric cancer and also CRC compared to those who do not [183]. VacA, another virulence factor carried by some *H. pylori* strains have not yet been associated with CRC but appeared to be a key factor in the colonization and virulence of *H. pylori* [184]. Finally, some *H. pylori* strains carry the virulence factor *Helicobacter pylori* neutrophil-activating protein (HP-NAP), which has been found to promote the production of ROS by neutrophils [185]. Moreover, *H. pylori* has been shown to induce the secretion of several pro-inflammatory mediators such as TNF-α, IFN-γ, IL-1β, IL-6, and IL-8 by infected cells showing its contribution in inflammation-induced cancer [186]. 

### 5.7. Escherichia coli

*E. coli* is a gram-negative, aero-anaerobic, commensal bacterium that colonizes the human gut soon after birth. *E. coli* has a symbiotic relationship with the host and is not normally implied in diseases. However, some virulent strains of *E. coli* have acquired pathogenic characteristics that allow them to colonize the human gut and promote the occurrence of intra- and extra-intestinal diseases. These *E. coli* strains can be divided into eight pathotypes based on their pathogenic profiles: enteropathogenic *E. coli* (EPEC), enterohemorrhagic *E. coli* (EHEC), enteroinvasive *E. coli* (EIEC), enteroaggregative *E. coli* (EAEC), enterotoxigenic *E. coli* (ETEC), diffusely adherent *E. coli* (DAEC), adherent-invasive *E. coli* (AIEC) and Shiga toxin-producing enteroaggregative *E. coli* (STEAEC) [187]. *E. coli* strains are divided into four main phylogenetic groups A, B1, B2 and D, with fecal strains often belong to A and B1 groups, whereas the pathogenic strains carrying the virulence factors most frequently belong to B2 and D groups [188]. Some strains of the B2 and D groups are associated with chronic inflammatory intestinal diseases which are known to be risk factors for CRC [189,190]. An enrichment of *E. coli* strains mainly belonging to the B2 and D groups in CRC patients has been shown. Indeed, *E. coli* strains were found in 90% and 93% of patients with adenomas and carcinomas respectively, whereas only 3% of colonic biopsies from asymptomatic control subjects are positive for *E. coli* [191]. In 2004, mucosa-associated *E. coli* was found enriched in 70% of the 21 CRC patients compared to 42% of the 24 control biopsies [192]. Using adenocarcinomas and normal colonic mucosa from CRC patients, mucosa-associated *E. coli* was found in 50% of adenomas compared to 15% of normal mucosal samples [193]. More recent studies have confirmed the enrichment of *E. coli* in tumors and mucosa from CRC patients compared to the control subjects [49,194,195,196]. Interestingly, in CRC samples, studies have shown a high prevalence of *E. coli* strains that harbor virulence factors and produce toxins called cyclomodulins able to induce DNA damage and/or influence the cell cycle of eukaryotic cells and therefore affecting cellular proliferation, differentiation and apoptosis [193,194,197]. Interestingly, there is a correlation between poor prognostic factors for CRC (tumor-node-metastasis stage) and colonization of mucosa with *E. coli* [196]. Cyclomodulin-producing *E. coli* strains are more prevalent on mucosa of patients with advanced stage III/IV CRC compared to those with stage I CRC, suggesting that pathogenic *E. coli* colonization could be used as a new and crucial prognostic marker [196]. Four toxins have been extensively studied for their impacts on CRC: CIF (cycle-inhibiting factor), CNF (cytotoxic necrotizing factor), CDT (cytolethal distending toxin) and colibactin. CIF is produced by certain EPEC strains, promotes the actin cytoskeleton rearrangement and mediates the G2/M cell cycle arrest characterized by inactive phosphorylation of cyclin-dependent kinase 1, a key player in cell cycle regulation [198]. CNF induces a transient activation of COX-2 and the Rho GTPases such as Rac, RhoA, and Cdc42. As Rho GTPases have been characterized as regulators of actin cytoskeleton, their deregulation leads to cytoskeletal alterations and therefore affects the cell cycle [199,200]. CDT was first identified in 1988 in the culture of *E. coli* strains isolated from patients with diarrhea. This toxin has been found in various gram-negative bacterial species and is known to have DNAse activity and therefore induce DNA double-strand breaks, cell cycle arrest and cell apoptosis if the DNA double-strand breaks exceed the repair capacity of the cell [200]. Colibactin is another bacterial-derived genotoxin first described in 2006 by Nougayrede and colleagues [201] and has not yet been isolated or purified to date. Colibactin is a hybride polyketide-non ribosomal peptide compound produced by a complex biosynthetic machinery encoded by the polyketide synthase (*pks*) pathogenicity island [201]. High prevalence of *E. coli* strains harboring the *pks* island has been associated with CRC [49,194]. In vitro, colibactin induces DNA double-strand breaks in eukaryotic cells with activation of the DNA damage signaling cascade and cell cycle arrest [201]. In addition, colibactin is able to induce chromosomic instability with sign of chromosome aberration [202]. In 2015, Vizcaino and Crawford were successful in purifying a pre-colibactin compound and showed that the pre-colibactin is able to induce in vitro DNA crosslink but not DNA double-strand breaks [203]. The authors thus hypothesized that DNA double-strand breaks may not be induced directly by colibactin but rather a response of infected mammalian cells to repair their DNA [203]. Experiments using human epithelial cells have shown that *pks*-harboring *E. coli* strains are able to induce senescence of infected cells, which is accompanied with ROS production, release of pro-inflammatory mediators and also production of growth factors, such as the hepatocyte growth factor, which have the ability to promote the proliferation of neighboring uninfected cells [204,205]. Using macrophages, which are one of the predominant tumor-infiltrating immune cells, Raisch and colleagues showed that *pks*-harboring *E. coli* strains are able to survive in macrophages and induce pro-inflammatory and pro-carcinogenic mediators such as COX-2 and PGE2 [206]. This suggests that *E. coli* might influence CRC progression by persisting in immune cells and controlling the secretion of pro-tumoral mediators [206]. Using a genetically modified mouse model, the *pks*-harboring *E. coli* strain 11G5 isolated from CRC was shown to highly persist in the gut, induce colonic inflammation, epithelial damages and cellular proliferation [197]. Using inflammation-induced CRC model (AOM-treated *il10*^−/−^ mice), mono-colonization with *pks*-harboring *E. coli* strains leads to enhanced tumor multiplicity and invasion compared to mice colonized with the isogenic mutant defective for *pks* island and therefore not able to produce colibactin, or compared to uninfected mice [49]. The effect of *pks*-harboring *E. coli* strains to enhance intestinal tumorigenesis is confirmed using *APC^Min/+^* mice [196] or xenograft and AOM-DSS mouse models of CRC [205]. Recently, a clinical study on 88 CRC patients showed a significant increase in *E. coli* colonization in the MSI CRC phenotype [207]. However, colibactin-producing *E. coli* are more frequently found in microsatellite stable (MSS) CRC, suggesting that the involvement of *pks*-harboring *E. coli* in CRC may depend on the CRC phenotype [207].

## 6. Conclusions and Future Directions/Clinical Application

CRC is a multifactorial disease, of which several risk factors have been identified involving genetic and environment factors, lifestyle and gut microbiota. Usually treated with surgery, chemotherapy and radiotherapy with high toxicity and treatment resistance, it is essential to propose less harmful new therapeutic strategies for CRC. Since gut microbiota can contribute to colorectal carcinogenesis, strategies targeting the gut microbiota have been proposed to prevent and treat CRC. A potential strategy could be the supplementation of SCFAs, which have beneficial effects on the epithelial barrier functions and mucosal immune response, as well as anti-inflammatory and anti-carcinogenic activities [9]. Indeed, administration of SCFAs was shown to inhibit colonic inflammation and decrease cellular proliferation marker levels leading to reduced colon tumorigenesis in AOM-DSS-treated mice [208]. The bacteria-induced ROS could also be targeted as a strategy for CRC prevention. For example, it was shown that inhibition of polyamine catabolism, which leads to formation of ROS, leads to decreases in ETBF-induced proliferation, chronic inflammation and tumorigenesis in *APC^Min/+^* mice [209]. Recently, Cougnoux et al. showed that two compounds that bind to the active site of ClbP enzyme involved in the synthesis of colibactin are able to suppress colibactin-induced DNA damage both in vitro using human epithelial Hela cells and in vivo using a mouse colonic loop model [10]. Using human HCT116 colon cancer cells, the authors showed that the treatment of *pks*-harboring *E. coli* with these two compounds significantly inhibits *pks*-harboring *E. coli*-induced cellular senescence, which consequently suppresses hepatocyte growth factor secretion and proliferation of neighboring uninfected cell. These two compounds are also able to reduce tumor growth in xenograft and AOM-DSS CRC models by inhibiting *pks*-harboring *E. coli*-induced senescence, decreasing hepatocyte growth factor levels and cell proliferation [10]. This study showed that targeting colibactin production could be a strategy to prevent the emergence of CRC induced by *pks*-harboring *E. coli*. The direct modulation of the gut microbiota is a highly considered strategy for CRC treatment. In this regard, two prebiotics, substances that induce the growth or activity of microorganisms and therefore positively influence the gut microbiota, galacto-oligosaccharide and inulin were shown to inhibit aberrant crypt foci formation [11]. In addition, inulin was shown to decrease carcinogen-induced DNA damage in intestinal crypts in mice [210]. Several probiotics have also been shown to have a great impact on prevention of CRC development. Indeed, the consumption of lactic acid bacteria-containing probiotics can prevent DNA damage induced by the mutagenic and carcinogenic heterocyclic amines [12]. Recently, *Lactobacillus* was shown to induce apoptosis of the human colorectal adenocarcinoma cell line HT29 by enhancing pro-apoptotic BAX protein expression and decreasing anti-apoptotic BCL-2 protein expression, leading to the inhibition of cell growth [211]. Using a mouse model of 1,2-dimethylhydazine-induced CRC, a *Lactobacillus* strain was shown to decrease the damage score and the number of colonic tumors [212]. Interestingly, the administration of a *Lactobacillus* strain induces expression of an anti-inflammatory cytokine profile with enhanced IL-10 level [212]. These studies suggest that the lactic acid-producing bacteria could be used to inhibit the inflammatory environment associated with CRC, and in a larger extent, to prevent the development of CRC in patients with chronic intestinal inflammation who have a high risk to develop CRC.

Microbiota composition has been shown to influence the response to chemotherapy or immunotherapy [213,214]. In 2015, Sivan and colleagues analyzed melanoma growth in mice from different animal facilities which have different commensal microbes [13]. They found a significant difference in tumor development, and this is immune-mediated with decreased tumor-specific T cell response and less CD8^+^ T cell accumulation in the tumors from mice with more aggressive tumor development. Cohousing ablates the difference in tumor growth between the mice from different facilities, showing the presence of commensal microbes that facilitate anti-tumor immunity [13]. By analyzing the bacterial community in fecal samples, the authors showed a positive association between *Bifidobacterium* and anti-tumor T cell response. Administration of *Bifidobacterium* significantly improves the control of tumor development in mice compared to untreated mice, and this is accompanied by an induction of tumor-specific T cells and increased accumulation of antigen-specific CD8^+^ T cells in the tumor. Of note, *Bifidobacterium* improves response to anti-programmed death-ligand 1 monoclonal antibody therapy, which is an anti-tumor immunotherapy, in mice [13]. Vétizou and colleagues have studied another anti-tumor immunotherapy, which relies on the blockade of cytotoxic T lymphocyte-associated antigen 4 (CTLA4), a major negative regulator of T cell activation against a variety of antigens including tumor-associated antigens. The authors showed that the CTLA4 immunotherapy is influenced by *Bacteroides* which stimulates the T cell response [14]. These results showed that the heterogeneity between patients in the response to anti-tumor immunotherapy is largely associated with the gut microbiota composition, suggesting that manipulation of gut microbiota could improve immunotherapy responses. 

Finally, immune therapies targeting TLRs to activate anti-cancer immunity or suppress oncogenic signaling pathways should be considered for CRC treatment. Various molecules targeting TLRs are currently under investigation in clinical trials for their ability to promote antitumor immunity [215]. For example, TLR9 agonists, which have already been added to anti-cancer strategies such as chemotherapy, radiotherapy and immunotherapy, are able to enhance the anti-tumor immune response mediated by T and B cells. Moreover, TLR9 agonists were shown to inhibit colon cancer cell proliferation, promote apoptosis, and improve the beneficial effects of radiotherapy [215]. Strategies using TLR4 antagonists have been also proposed in CRC treatment. Indeed, anti-TLR4 antibodies have been shown to decrease the number of polyps in AOM-DSS-treated mice [216]. 

In conclusion, the modulation of the gut microbiota by all the strategies outlined here can have a beneficial impact on the dialogue between the gut, the immune system and the microbiota. In addition, increasing evidence shows that gut microbiota manipulation can exert a protective effect against CRC via the production of SCFAs, inhibition of toxin-producing pathogens, anti-proliferative activity, reduction of aberrant crypt foci and enhanced production of anti-oxidant enzymes and anti-inflammatory responses. Moreover, the identification of other microbes associated with clinical benefits or microbes as biomarkers to predict immunotherapy response should be considered.

## Abbreviations

AIEC	Adherent-invasive *Escherichia coli*
AKT	Protein kinase B
AMPs	Antimicrobial peptides
AOM	Azoxymethane
Apc	Adenomatous polyposis coli
BAX	Bcl-2-associated X
BCL-2	B-cell lymphoma 2
BfPAI	*Bacteroides fragilis* pathogenicity island
CDT	Cytolethal distending toxin
CIF	Cycle-inhibiting factor
CIMP	CpG island methylator phenotype
CIN	Chromosomal instability
CLRs	C-type lectin receptors
CNF	Cytotoxic necrotizing factor
COX-2	Cyclo-oxygénase-2
CRC	Colorectal cancer
CTLA4	Cytotoxic T lymphocyte-associated antigen 4
DAEC	Diffusely adherent *Escherichia coli*
DSS	Dextran sodium sulfate
EAEC	Enteroaggregative *Escherichia coli*
EHEC	Enterohemorrhagic *Escherichia coli*
EIEC	Enteroinvasive *Escherichia coli*
EPEC	Enteropathogenic *Escherichia coli*
ETBF	Enterotoxigenic *Bacteroides fragilis*
ETEC	Enterotoxigenic *Escherichia coli*
FAP	Familial adenomatous polyposis
GM-CSF	Granulocyte macrophage colony-stimulating factor
HP-NAP	*Helicobacter pylori* neutrophil-activating protein
IBD	Inflammatory bowel diseases
IECs	Intestinal epithelial cells
IgA	Immunoglobulin-A
IL	Interleukin
ILC3	Innate lymphoid cells
IFN-γ	Interferon gamma
M cells	Microfold cells
MALT	Mucosa-associated lymphoid tissue
MAMPs	Microbe-associated molecular patterns
MAPK	Mitogen-activated protein kinase
MLH1	MutL homolog 1
MSI	Microsatellite instability
mTOR	Mammalian target of rapamycin
MyD88	Myeloid differentiation primary response gene 88
NF-κB	Nuclear factor-kappa B
NLRs	NOD-like receptors
NOD	Nucleotide-binding oligomerization domain
NTBF	Nontoxigenic *Bacteroides fragilis*
PAMPs	Pathogen-associated molecular patterns
PGE2	Prostaglandin E2
PI3K	Phosphoinositide 3-kinase
*pks*	Polyketide synthase
PRRs	Pattern recognition receptors
ROS	Reactive oxygen species
RNS	Reactive nitrogen species
SCFAs	Short-chain fatty acids
SFB	Segmented filamentous bacteria
SNP	Single nucleotide polymorphism
STAT3	Signal transducer and activator of transcription 3
STEAEC	Shiga toxin-producing enteroaggregative *Escherichia coli*
TCF	T-cell factor
Th17	T helper 17
TLRs	Toll-like receptors
TNF-α	Tumor necrosis factor-α

## References

[1] Ferlay J., Soerjomataram I., Dikshit R., Eser S., Mathers C., Rebelo M., Parkin D.M., Forman D., Bray F. (2015). Cancer incidence and mortality worldwide: Sources, methods and major patterns in GLOBOCAN 2012. Int. J. Cancer.

[2] Terzić J., Grivennikov S., Karin E., Karin M. (2010). Inflammation and colon cancer. Gastroenterology.

[3] Brenner H., Kloor M., Pox C.P. (2014). Colorectal cancer. Lancet.

[4] Tjalsma H., Boleij A., Marchesi J.R., Dutilh B.E. (2012). A bacterial driver–passenger model for colorectal cancer: Beyond the usual suspects. Nat. Rev. Microbiol..

[5] Chen W., Liu F., Ling Z., Tong X., Xiang C. (2012). Human intestinal lumen and mucosa-associated microbiota in patients with colorectal cancer. PLoS ONE.

[6] Lu Y., Chen J., Zheng J., Hu G., Wang J., Huang C., Lou L., Wang X., Zeng Y. (2016). Mucosal adherent bacterial dysbiosis in patients with colorectal adenomas. Sci. Rep..

[7] Gao Z., Guo B., Gao R., Zhu Q., Qin H. (2015). Microbiota disbiosis is associated with colorectal cancer. Front. Microbiol..

[8] Schwabe R.F., Jobin C. (2013). The microbiome and cancer. Nat. Rev. Cancer.

[9] Van der Beek C.M., Dejong C.H.C., Troost F.J., Masclee A.A.M., Lenaerts K. (2017). Role of short-chain fatty acids in colonic inflammation, carcinogenesis, and mucosal protection and healing. Nutr. Rev..

[10] Cougnoux A., Delmas J., Gibold L., Faïs T., Romagnoli C., Robin F., Cuevas-Ramos G., Oswald E., Darfeuille-Michaud A., Prati F. (2016). Small-molecule inhibitors prevent the genotoxic and protumoural effects induced by colibactin-producing bacteria. Gut.

[11] Qamar T.R., Syed F., Nasir M., Rehman H., Zahid M.N., Liu R.H., Iqbal S. (2016). Novel Combination of Prebiotics Galacto-Oligosaccharides and Inulin-Inhibited Aberrant Crypt Foci Formation and Biomarkers of Colon Cancer in Wistar Rats. Nutrients.

[12] Zsivkovits M., Fekadu K., Sontag G., Nabinger U., Huber W.W., Kundi M., Chakraborty A., Foissy H., Knasmüller S. (2003). Prevention of heterocyclic amine-induced DNA damage in colon and liver of rats by different lactobacillus strains. Carcinogenesis.

[13] Sivan A., Corrales L., Hubert N., Williams J.B., Aquino-Michaels K., Earley Z.M., Benyamin F.W., Lei Y.M., Jabri B., Alegre M.-L. (2015). Commensal Bifidobacterium promotes antitumor immunity and facilitates anti-PD-L1 efficacy. Science.

[14] Vétizou M., Pitt J.M., Daillère R., Lepage P., Waldschmitt N., Flament C., Rusakiewicz S., Routy B., Roberti M.P., Duong C.P.M. (2015). Anticancer immunotherapy by CTLA-4 blockade relies on the gut microbiota. Science.

[15] Johns L.E., Houlston R.S. (2001). A systematic review and meta-analysis of familial colorectal cancer risk. Am. J. Gastroenterol..

[16] Jasperson K.W., Tuohy T.M., Neklason D.W., Burt R.W. (2010). Hereditary and familial colon cancer. Gastroenterology.

[17] Doubeni C.A., Laiyemo A.O., Major J.M., Schootman M., Lian M., Park Y., Graubard B.I., Hollenbeck A.R., Sinha R. (2012). Socioeconomic status and the risk of colorectal cancer: An analysis of over one-half million adults in the NIH-AARP Diet and Health Study. Cancer.

[18] Ryan-Harshman M., Aldoori W. (2007). Diet and colorectal cancer. Can. Fam. Physician.

[19] Boffetta P., Hashibe M. (2006). Alcohol and cancer. Lancet Oncol..

[20] Cho E., Smith-Warner S.A., Ritz J., van den Brandt P.A., Colditz G.A., Folsom A.R., Freudenheim J.L., Giovannucci E., Goldbohm R.A., Graham S. (2004). Alcohol intake and colorectal cancer: A pooled analysis of 8 cohort studies. Ann. Intern. Med..

[21] Botteri E., Iodice S., Bagnardi V., Raimondi S., Lowenfels A.B., Maisonneuve P. (2008). Smoking and colorectal cancer: A meta-analysis. JAMA.

[22] Berger N.A. (2014). Obesity and cancer pathogenesis. Ann. N. Y. Acad. Sci..

[23] Ma Y., Yang Y., Wang F., Zhang P., Shi C., Zou Y., Qin H. (2013). Obesity and Risk of Colorectal Cancer: A Systematic Review of Prospective Studies. PLoS ONE.

[24] Lin J.E., Colon-Gonzalez F., Blomain E., Kim G.W., Aing A., Stoecker B., Rock J., Snook A.E., Zhan T., Hyslop T.M. (2016). Obesity-induced colorectal cancer is driven by caloric silencing of the guanylin-GUCY2C paracrine signaling axis. Cancer Res..

[25] Koda M., Sulkowska M., Kanczuga-Koda L., Surmacz E., Sulkowski S. (2007). Overexpression of the obesity hormone leptin in human colorectal cancer. J. Clin. Pathol..

[26] Wang D., Chen J., Chen H., Duan Z., Xu Q., Wei M., Wang L., Zhong M. (2012). Leptin regulates proliferation and apoptosis of colorectal carcinoma through PI3K/Akt/mTOR signalling pathway. J. Biosci..

[27] Walter V., Jansen L., Knebel P., Chang-Claude J., Hoffmeister M., Brenner H. (2017). Physical activity and survival of colorectal cancer patients: Population-based study from Germany. Int. J. Cancer.

[28] Golshiri P., Rasooli S., Emami M., Najimi A. (2016). Effects of Physical Activity on Risk of Colorectal Cancer: A Case-control Study. Int. J. Prev. Med..

[29] Ghafari M., Mohammadian M., Valipour A.A., Mohammadian-Hafshejani A. (2016). Physical Activity and Colorectal Cancer. Iran. J. Public Health.

[30] Kruk J., Czerniak U. (2013). Physical activity and its relation to cancer risk: Updating the evidence. Asian Pac. J. Cancer Prev..

[31] Jess T., Rungoe C., Peyrin-Biroulet L. (2012). Risk of colorectal cancer in patients with ulcerative colitis: A meta-analysis of population-based cohort studies. Clin. Gastroenterol. Hepatol..

[32] Farraye F.A., Odze R.D., Eaden J., Itzkowitz S.H. (2010). AGA Medical Position Statement on the Diagnosis and Management of Colorectal Neoplasia in Inflammatory Bowel Disease. Gastroenterology.

[33] Haslam A., Robb S.W., Hébert J.R., Huang H., Wirth M.D., Shivappa N., Ebell M.H. (2017). The association between Dietary Inflammatory Index scores and the prevalence of colorectal adenoma. Public Health Nutr..

[34] Gupta R.A., Dubois R.N. (2001). Colorectal cancer prevention and treatment by inhibition of cyclooxygenase-2. Nat. Rev. Cancer.

[35] Tanaka T., Kohno H., Suzuki R., Hata K., Sugie S., Niho N., Sakano K., Takahashi M., Wakabayashi K. (2006). Dextran sodium sulfate strongly promotes colorectal carcinogenesis in *Apc^Min/+^* mice: Inflammatory stimuli by dextran sodium sulfate results in development of multiple colonic neoplasms. Int. J. Cancer.

[36] Neufert C., Becker C., Neurath M.F. (2007). An inducible mouse model of colon carcinogenesis for the analysis of sporadic and inflammation-driven tumor progression. Nat. Protoc..

[37] Bayarsaihan D. (2011). Epigenetic Mechanisms in Inflammation. J. Dent. Res..

[38] Meira L.B., Bugni J.M., Green S.L., Lee C.-W., Pang B., Borenshtein D., Rickman B.H., Rogers A.B., Moroski-Erkul C.A., McFaline J.L. (2008). DNA damage induced by chronic inflammation contributes to colon carcinogenesis in mice. J. Clin. Investig..

[39] Francescone R., Hou V., Grivennikov S.I. (2015). Cytokines, IBD and colitis-associated cancer. Inflamm. Bowel Dis..

[40] Basavaraju U., Shebl F.M., Palmer A.J., Berry S., Hold G.L., El-Omar E.M., Rabkin C.S. (2015). Cytokine gene polymorphisms, cytokine levels and the risk of colorectal neoplasia in a screened population of Northeast Scotland. Eur. J. Cancer Prev..

[41] Kim S., Keku T.O., Martin C., Galanko J., Woosley J.T., Schroeder J.C., Satia J.A., Halabi S., Sandler R.S. (2008). Circulating levels of inflammatory cytokines and risk of colorectal adenomas. Cancer Res..

[42] Song M., Mehta R.S., Wu K., Fuchs C.S., Ogino S., Giovannucci E.L., Chan A.T. (2016). Plasma Inflammatory Markers and Risk of Advanced Colorectal Adenoma in Women. Cancer Prev. Res..

[43] Knüpfer H., Preiss R. (2010). Serum interleukin-6 levels in colorectal cancer patients—A summary of published results. Int. J. Colorectal Dis..

[44] Hsu C.-P., Chung Y.-C. (2006). Influence of interleukin-6 on the invasiveness of human colorectal carcinoma. Anticancer Res..

[45] Grivennikov S., Karin E., Terzic J., Mucida D., Yu G.-Y., Vallabhapurapu S., Scheller J., Rose-John S., Cheroutre H., Eckmann L. (2009). IL-6 and Stat3 are required for survival of intestinal epithelial cells and development of colitis-associated cancer. Cancer Cell.

[46] Kühn R., Löhler J., Rennick D., Rajewsky K., Müller W. (1993). Interleukin-10-deficient mice develop chronic enterocolitis. Cell.

[47] Uronis J.M., Mühlbauer M., Herfarth H.H., Rubinas T.C., Jones G.S., Jobin C. (2009). Modulation of the Intestinal Microbiota Alters Colitis-Associated Colorectal Cancer Susceptibility. PLoS ONE.

[48] Tomkovich S., Yang Y., Winglee K., Gauthier J., Mühlbauer M., Sun X., Mohamadzadeh M., Liu X., Martin P., Wang G.P. (2017). Locoregional Effects of Microbiota in a Preclinical Model of Colon Carcinogenesis. Cancer Res..

[49] Arthur J.C., Perez-Chanona E., Mühlbauer M., Tomkovich S., Uronis J.M., Fan T.-J., Campbell B.J., Abujamel T., Dogan B., Rogers A.B. (2012). Intestinal inflammation targets cancer-inducing activity of the microbiota. Science.

[50] Medvedev A.E. (2013). Toll-like receptor polymorphisms, inflammatory and infectious diseases, allergies, and cancer. J. Interferon Cytokine Res..

[51] Weber A.N., Försti A. (2014). Toll-like receptor genetic variants and colorectal cancer. Oncoimmunology..

[52] Semlali A., Parine N.R., Al Amri A., Azzi A., Arafah M., Kohailan M., Shaik J.P., Almadi M.A., Aljebreen A.M., Alharbi O. (2016). Association between TLR-9 polymorphisms and colon cancer susceptibility in Saudi Arabian female patients. OncoTargets Ther..

[53] Sipos F., Fűri I., Constantinovits M., Tulassay Z., Műzes G. (2014). Contribution of TLR signaling to the pathogenesis of colitis-associated cancer in inflammatory bowel disease. World J. Gastroenterol..

[54] Peng G., Guo Z., Kiniwa Y., Voo K.S., Peng W., Fu T., Wang D.Y., Li Y., Wang H.Y., Wang R.-F. (2005). Toll-like receptor 8-mediated reversal of CD4+ regulatory T cell function. Science.

[55] Hoon Rhee S., Im E., Pothoulakis C. (2008). Toll-like receptor 5 engagement modulates tumor development and growth in a mouse xenograft model of human colon cancer. Gastroenterology.

[56] Heckelsmiller K., Beck S., Rall K., Sipos B., Schlamp A., Tuma E., Rothenfusser S., Endres S., Hartmann G. (2002). Combined dendritic cell- and CpG oligonucleotide-based immune therapy cures large murine tumors that resist chemotherapy. Eur. J. Immunol..

[57] Lowe E.L., Crother T.R., Rabizadeh S., Hu B., Wang H., Chen S., Shimada K., Wong M.H., Michelsen K.S., Arditi M. (2010). Toll-Like Receptor 2 Signaling Protects Mice from Tumor Development in a Mouse Model of Colitis-Induced Cancer. PLoS ONE.

[58] Huang B., Zhao J., Li H., He K.-L., Chen Y., Mayer L., Unkeless J.C., Xiong H. (2005). Toll-Like Receptors on Tumor Cells Facilitate Evasion of Immune Surveillance. Cancer Res..

[59] Fukata M., Hernandez Y., Conduah D., Cohen J., Chen A., Breglio K., Goo T., Hsu D., Xu R., Abreu M.T. (2009). Innate immune signaling by Toll-like receptor-4 (TLR4) shapes the inflammatory microenvironment in colitis-associated tumors. Inflamm. Bowel Dis..

[60] Rakoff-Nahoum S., Medzhitov R. (2007). Regulation of spontaneous intestinal tumorigenesis through the adaptor protein MyD88. Science.

[61] Kurzawski G., Suchy J., Kładny J., Grabowska E., Mierzejewski M., Jakubowska A., Debniak T., Cybulski C., Kowalska E., Szych Z. (2004). The NOD2 3020insC mutation and the risk of colorectal cancer. Cancer Res..

[62] Philpott D.J., Sorbara M.T., Robertson S.J., Croitoru K., Girardin S.E. (2014). NOD proteins: Regulators of inflammation in health and disease. Nat. Rev. Immunol..

[63] Zhan Y., Seregin S.S., Chen J., Chen G.Y. (2016). Nod1 Limits Colitis-Associated Tumorigenesis by Regulating IFN-γ Production. J. Immunol..

[64] Qin J., Li R., Raes J., Arumugam M., Burgdorf K.S., Manichanh C., Nielsen T., Pons N., Levenez F., Yamada T. (2010). A human gut microbial gene catalogue established by metagenomic sequencing. Nature.

[65] Dominguez-Bello M.G., Blaser M.J., Ley R.E., Knight R. (2011). Development of the human gastrointestinal microbiota and insights from high-throughput sequencing. Gastroenterology.

[66] Jandhyala S.M., Talukdar R., Subramanyam C., Vuyyuru H., Sasikala M., Reddy D.N. (2015). Role of the normal gut microbiota. World J. Gastroenterol..

[67] Sun Y., O’Riordan M.X.D. (2013). Regulation of Bacterial Pathogenesis by Intestinal Short-Chain Fatty Acids. Adv. Appl. Microbiol..

[68] Rowland I., Gibson G., Heinken A., Scott K., Swann J., Thiele I., Tuohy K. (2017). Gut microbiota functions: Metabolism of nutrients and other food components. Eur. J. Nutr..

[69] Woting A., Blaut M. (2016). The Intestinal Microbiota in Metabolic Disease. Nutrients.

[70] Vernocchi P., Del Chierico F., Putignani L. (2016). Gut Microbiota Profiling: Metabolomics Based Approach to Unravel Compounds Affecting Human Health. Front. Microbiol..

[71] Fukiya S., Arata M., Kawashima H., Yoshida D., Kaneko M., Minamida K., Watanabe J., Ogura Y., Uchida K., Itoh K. (2009). Conversion of cholic acid and chenodeoxycholic acid into their 7-oxo derivatives by Bacteroides intestinalis AM-1 isolated from human feces. FEMS Microbiol. Lett..

[72] Ajouz H., Mukherji D., Shamseddine A. (2014). Secondary bile acids: An underrecognized cause of colon cancer. World J. Surg. Oncol..

[73] Neis E.P., Dejong C.H., Rensen S.S. (2015). The Role of Microbial Amino Acid Metabolism in Host Metabolism. Nutrients.

[74] Hill M.J. (1997). Intestinal flora and endogenous vitamin synthesis. Eur. J. Cancer Prev..

[75] Frick P.G., Riedler G., Brögli H. (1967). Dose response and minimal daily requirement for vitamin K in man. J. Appl. Physiol..

[76] Gustafsson B.E., Daft F.S., McDaniel E.G., Smith J.C., Fitzgerald R.J. (1962). Effects of vitamin K-active compounds and intestinal microorganisms in vitamin K-deficient germfree rats. J. Nutr..

[77] Johansson M.E.V., Phillipson M., Petersson J., Velcich A., Holm L., Hansson G.C. (2008). The inner of the two Muc2 mucin-dependent mucus layers in colon is devoid of bacteria. Proc. Natl. Acad. Sci. USA.

[78] Wang H.-B., Wang P.-Y., Wang X., Wan Y.-L., Liu Y.-C. (2012). Butyrate Enhances Intestinal Epithelial Barrier Function via Up-Regulation of Tight Junction Protein Claudin-1 Transcription. Dig. Dis. Sci..

[79] Gaudier E., Jarry A., Blottière H.M., de Coppet P., Buisine M.P., Aubert J.P., Laboisse C., Cherbut C., Hoebler C. (2004). Butyrate specifically modulates MUC gene expression in intestinal epithelial goblet cells deprived of glucose. Am. J. Physiol. Gastrointest. Liver Physiol..

[80] Hernández-Chirlaque C., Aranda C.J., Ocón B., Capitán-Cañadas F., Ortega-González M., Carrero J.J., Suárez M.D., Zarzuelo A., Sánchez de Medina F., Martínez-Augustin O. (2016). Germ-free and Antibiotic-treated Mice are Highly Susceptible to Epithelial Injury in DSS Colitis. J. Crohns. Colitis.

[81] Bain C.C., Mowat A.M. (2014). Macrophages in intestinal homeostasis and inflammation. Immunol. Rev..

[82] Zindl C.L., Lai J.-F., Lee Y.K., Maynard C.L., Harbour S.N., Ouyang W., Chaplin D.D., Weaver C.T. (2013). IL-22-producing neutrophils contribute to antimicrobial defense and restitution of colonic epithelial integrity during colitis. Proc. Natl. Acad. Sci. USA.

[83] Chu V.T., Beller A., Rausch S., Strandmark J., Zänker M., Arbach O., Kruglov A., Berek C. (2014). Eosinophils promote generation and maintenance of immunoglobulin-A-expressing plasma cells and contribute to gut immune homeostasis. Immunity.

[84] Sonnenberg G.F., Artis D. (2015). Innate lymphoid cells in the initiation, regulation and resolution of inflammation. Nat. Med..

[85] Mortha A., Chudnovskiy A., Hashimoto D., Bogunovic M., Spencer S.P., Belkaid Y., Merad M. (2014). Microbiota-dependent crosstalk between macrophages and ILC3 promotes intestinal homeostasis. Science.

[86] Hepworth M.R., Monticelli L.A., Fung T.C., Ziegler C.G.K., Grunberg S., Sinha R., Mantegazza A.R., Ma H.-L., Crawford A., Angelosanto J.M. (2013). Innate lymphoid cells regulate CD4+ T-cell responses to intestinal commensal bacteria. Nature.

[87] Bekiaris V., Persson E.K., Agace W.W. (2014). Intestinal dendritic cells in the regulation of mucosal immunity. Immunol. Rev..

[88] Slack E., Balmer M.L., Macpherson A.J. (2014). B cells as a critical node in the microbiota-host immune system network. Immunol. Rev..

[89] Yamanaka T., Helgeland L., Farstad I.N., Fukushima H., Midtvedt T., Brandtzaeg P. (2003). Microbial colonization drives lymphocyte accumulation and differentiation in the follicle-associated epithelium of Peyer’s patches. J. Immunol..

[90] Macpherson A.J., Harris N.L. (2004). Interactions between commensal intestinal bacteria and the immune system. Nat. Rev. Immunol..

[91] Ivanov I.I., Atarashi K., Manel N., Brodie E.L., Shima T., Karaoz U., Wei D., Goldfarb K.C., Santee C.A., Lynch S.V. (2009). Induction of intestinal Th17 cells by segmented filamentous bacteria. Cell.

[92] Cao A.T., Yao S., Gong B., Elson C.O., Cong Y. (2012). Th17 cells upregulate polymeric Ig receptor and intestinal IgA and contribute to intestinal homeostasis. J. Immunol..

[93] Cerf-Bensussan N., Gaboriau-Routhiau V. (2010). The immune system and the gut microbiota: Friends or foes?. Nat. Rev. Immunol..

[94] Schnupf P., Gaboriau-Routhiau V., Gros M., Friedman R., Moya-Nilges M., Nigro G., Cerf-Bensussan N., Sansonetti P.J. (2015). Growth and host interaction of mouse segmented filamentous bacteria in vitro. Nature.

[95] Gaboriau-Routhiau V., Rakotobe S., Lécuyer E., Mulder I., Lan A., Bridonneau C., Rochet V., Pisi A., De Paepe M., Brandi G. (2009). The key role of segmented filamentous bacteria in the coordinated maturation of gut helper T cell responses. Immunity.

[96] Macpherson A.J., Smith K. (2006). Mesenteric lymph nodes at the center of immune anatomy. J. Exp. Med..

[97] Arpaia N., Campbell C., Fan X., Dikiy S., van der Veeken J., deRoos P., Liu H., Cross J.R., Pfeffer K., Coffer P.J. (2013). Metabolites produced by commensal bacteria promote peripheral regulatory T-cell generation. Nature.

[98] Burgess S.L., Buonomo E., Carey M., Cowardin C., Naylor C., Noor Z., Wills-Karp M., Petri W.A. (2014). Bone marrow dendritic cells from mice with an altered microbiota provide interleukin 17A-dependent protection against Entamoeba histolytica colitis. MBio.

[99] Bammann L.L., Clark W.B., Gibbons R.J. (1978). Impaired colonization of gnotobiotic and conventional rats by streptomycin-resistant strains of Streptococcus mutans. Infect. Immun..

[100] Rupnik M., Wilcox M.H., Gerding D.N. (2009). Clostridium difficile infection: New developments in epidemiology and pathogenesis. Nat. Rev. Microbiol..

[101] Boleij A., Tjalsma H. (2012). Gut bacteria in health and disease: A survey on the interface between intestinal microbiology and colorectal cancer. Biol. Rev. Camb. Philos. Soc..

[102] Schamberger G.P., Diez-Gonzalez F. (2002). Selection of recently isolated colicinogenic Escherichia coli strains inhibitory to Escherichia coli O157:H7. J. Food Prot..

[103] Fukuda S., Toh H., Hase K., Oshima K., Nakanishi Y., Yoshimura K., Tobe T., Clarke J.M., Topping D.L., Suzuki T. (2011). Bifidobacteria can protect from enteropathogenic infection through production of acetate. Nature.

[104] Gantois I., Ducatelle R., Pasmans F., Haesebrouck F., Hautefort I., Thompson A., Hinton J.C., Van Immerseel F. (2006). Butyrate Specifically Down-Regulates Salmonella Pathogenicity Island 1 Gene Expression. Appl. Environ. Microbiol..

[105] Momose Y., Hirayama K., Itoh K. (2008). Competition for proline between indigenous Escherichia coli and *E. coli* O157:H7 in gnotobiotic mice associated with infant intestinal microbiota and its contribution to the colonization resistance against *E. coli* O157:H7. Antonie Van Leeuwenhoek.

[106] Marteyn B., West N., Browning D., Cole J., Shaw J., Palm F., Mounier J., Prévost M.-C., Sansonetti P., Tang C. (2010). Modulation of Shigella virulence in response to available oxygen in vivo. Nature.

[107] Takeuchi O., Akira S. (2010). Pattern recognition receptors and inflammation. Cell.

[108] Salzman N.H., Underwood M.A., Bevins C.L. (2007). Paneth cells, defensins, and the commensal microbiota: A hypothesis on intimate interplay at the intestinal mucosa. Semin. Immunol..

[109] Vaishnava S., Behrendt C.L., Ismail A.S., Eckmann L., Hooper L.V. (2008). Paneth cells directly sense gut commensals and maintain homeostasis at the intestinal host-microbial interface. Proc. Natl. Acad. Sci. USA.

[110] Kobayashi K.S., Chamaillard M., Ogura Y., Henegariu O., Inohara N., Nuñez G., Flavell R.A. (2005). Nod2-Dependent Regulation of Innate and Adaptive Immunity in the Intestinal Tract. Science.

[111] Frantz A.L., Rogier E.W., Weber C.R., Shen L., Cohen D.A., Fenton L.A., Bruno M.E. C., Kaetzel C.S. (2012). Targeted deletion of MyD88 in intestinal epithelial cells results in compromised antibacterial immunity associated with downregulation of polymeric immunoglobulin receptor, mucin-2, and antibacterial peptides. Mucosal Immunol..

[112] Cash H.L., Whitham C.V., Behrendt C.L., Hooper L.V. (2006). Symbiotic Bacteria Direct Expression of an Intestinal Bactericidal Lectin. Science.

[113] Giel J.L., Sorg J.A., Sonenshein A.L., Zhu J. (2010). Metabolism of Bile Salts in Mice Influences Spore Germination in Clostridium difficile. PLoS ONE.

[114] Plummer M., de Martel C., Vignat J., Ferlay J., Bray F., Franceschi S. (2016). Global burden of cancers attributable to infections in 2012: A synthetic analysis. Lancet Glob. Health.

[115] Schottenfeld D., Beebe-Dimmer J. (2015). The cancer burden attributable to biologic agents. Ann. Epidemiol..

[116] Moore W.E., Moore L.H. (1995). Intestinal floras of populations that have a high risk of colon cancer. Appl. Environ. Microbiol..

[117] Wang T., Cai G., Qiu Y., Fei N., Zhang M., Pang X., Jia W., Cai S., Zhao L. (2012). Structural segregation of gut microbiota between colorectal cancer patients and healthy volunteers. ISME J..

[118] Sobhani I., Tap J., Roudot-Thoraval F., Roperch J.P., Letulle S., Langella P., Corthier G., Tran Van Nhieu J., Furet J.P. (2011). Microbial dysbiosis in colorectal cancer (CRC) patients. PLoS ONE.

[119] Shen X.J., Rawls J.F., Randall T., Burcal L., Mpande C.N., Jenkins N., Jovov B., Abdo Z., Sandler R.S., Keku T.O. (2010). Molecular characterization of mucosal adherent bacteria and associations with colorectal adenomas. Gut Microbes.

[120] McCoy A.N., Araújo-Pérez F., Azcárate-Peril A., Yeh J.J., Sandler R.S., Keku T.O. (2013). Fusobacterium Is Associated with Colorectal Adenomas. PLoS ONE.

[121] Wu N., Yang X., Zhang R., Li J., Xiao X., Hu Y., Chen Y., Yang F., Lu N., Wang Z. (2013). Dysbiosis signature of fecal microbiota in colorectal cancer patients. Microb. Ecol..

[122] Viljoen K.S., Dakshinamurthy A., Goldberg P., Blackburn J.M. (2015). Quantitative profiling of colorectal cancer-associated bacteria reveals associations between fusobacterium spp., enterotoxigenic Bacteroides fragilis (ETBF) and clinicopathological features of colorectal cancer. PLoS ONE.

[123] Zackular J.P., Baxter N.T., Iverson K.D., Sadler W.D., Petrosino J.F., Chen G.Y., Schloss P.D. (2013). The gut microbiome modulates colon tumorigenesis. MBio.

[124] Morgan X.C., Tickle T.L., Sokol H., Gevers D., Devaney K.L., Ward D.V., Reyes J.A., Shah S.A., LeLeiko N., Snapper S.B. (2012). Dysfunction of the intestinal microbiome in inflammatory bowel disease and treatment. Genome Biol..

[125] Zhu Q., Jin Z., Wu W., Gao R., Guo B., Gao Z., Yang Y., Qin H. (2014). Analysis of the Intestinal Lumen Microbiota in an Animal Model of Colorectal Cancer. PLoS ONE.

[126] Strofilas A., Lagoudianakis E.E., Seretis C., Pappas A., Koronakis N., Keramidaris D., Koukoutsis I., Chrysikos I., Manouras I., Manouras A. (2012). Association of Helicobacter Pylori Infection and Colon Cancer. J. Clin. Med. Res..

[127] Balamurugan R., Rajendiran E., George S., Samuel G.V., Ramakrishna B.S. (2008). Real-time polymerase chain reaction quantification of specific butyrate-producing bacteria, Desulfovibrio and Enterococcus faecalis in the feces of patients with colorectal cancer. J. Gastroenterol. Hepatol..

[128] Zhou Y., He H., Xu H., Li Y., Li Z., Du Y., He J., Zhou Y., Wang H., Nie Y. (2016). Association of oncogenic bacteria with colorectal cancer in South China. Oncotarget.

[129] Amarnani R., Rapose A. (2017). Colon cancer and enterococcus bacteremia co-affection: A dangerous alliance. J. Infect. Public Health.

[130] Balish E., Warner T. (2002). Enterococcus faecalis induces inflammatory bowel disease in interleukin-10 knockout mice. Am. J. Pathol..

[131] Ruiz P.A., Shkoda A., Kim S.C., Sartor R.B., Haller D. (2005). IL-10 gene-deficient mice lack TGF-beta/Smad signaling and fail to inhibit proinflammatory gene expression in intestinal epithelial cells after the colonization with colitogenic Enterococcus faecalis. J. Immunol..

[132] Huycke M.M., Joyce W., Wack M.F. (1996). Augmented production of extracellular superoxide by blood isolates of Enterococcus faecalis. J. Infect. Dis..

[133] Huycke M.M., Abrams V., Moore D.R. (2002). Enterococcus faecalis produces extracellular superoxide and hydrogen peroxide that damages colonic epithelial cell DNA. Carcinogenesis.

[134] Limoli C.L., Giedzinski E. (2003). Induction of Chromosomal Instability by Chronic Oxidative Stress. Neoplasia.

[135] Wang X., Huycke M.M. (2007). Extracellular superoxide production by Enterococcus faecalis promotes chromosomal instability in mammalian cells. Gastroenterology.

[136] Wang X., Yang Y., Huycke M.M. (2015). Commensal bacteria drive endogenous transformation and tumour stem cell marker expression through a bystander effect. Gut.

[137] Zhang G., Svenungsson B., Kärnell A., Weintraub A. (1999). Prevalence of enterotoxigenic Bacteroides fragilis in adult patients with diarrhea and healthy controls. Clin. Infect. Dis..

[138] Sears C.L. (2001). The toxins of Bacteroides fragilis. Toxicon.

[139] Toprak N.U., Yagci A., Gulluoglu B.M., Akin M.L., Demirkalem P., Celenk T., Soyletir G. (2006). A possible role of Bacteroides fragilis enterotoxin in the aetiology of colorectal cancer. Clin. Microbiol. Infect..

[140] Boleij A., Hechenbleikner E.M., Goodwin A.C., Badani R., Stein E.M., Lazarev M.G., Ellis B., Carroll K.C., Albesiano E., Wick E.C. (2015). The Bacteroides fragilis toxin gene is prevalent in the colon mucosa of colorectal cancer patients. Clin. Infect. Dis..

[141] Purcell R.V., Pearson J., Aitchison A., Dixon L., Frizelle F.A., Keenan J.I. (2017). Colonization with enterotoxigenic Bacteroides fragilis is associated with early-stage colorectal neoplasia. PLoS ONE.

[142] Obiso R.J., Azghani A.O., Wilkins T.D. (1997). The Bacteroides fragilis toxin fragilysin disrupts the paracellular barrier of epithelial cells. Infect. Immun..

[143] Wells C., van de Westerlo E., Jechorek R., Feltis B., Wilkins T., Erlandsen S. (1996). Bacteroides fragilis enterotoxin modulates epithelial permeability and bacterial internalization by HT-29 enterocytes. Gastroenterology.

[144] Riegler M., Lotz M., Sears C., Pothoulakis C., Castagliuolo I., Wang C.C., Sedivy R., Sogukoglu T., Cosentini E., Bischof G. (1999). Bacteroides fragilis toxin 2 damages human colonic mucosa in vitro. Gut.

[145] Wu S., Morin P.J., Maouyo D., Sears C.L. (2003). Bacteroides fragilis enterotoxin induces c-Myc expression and cellular proliferation. Gastroenterology.

[146] Wu S., Rhee K.-J., Albesiano E., Rabizadeh S., Wu X., Yen H.-R., Huso D.L., Brancati F.L., Wick E., McAllister F. (2009). A human colonic commensal promotes colon tumorigenesis via activation of T helper type 17 T cell responses. Nat. Med..

[147] Han Y.W. (2015). Fusobacterium nucleatum: A commensal-turned pathogen. Curr. Opin. Microbiol..

[148] Castellarin M., Warren R.L., Freeman J.D., Dreolini L., Krzywinski M., Strauss J., Barnes R., Watson P., Allen-Vercoe E., Moore R.A. (2012). Fusobacterium nucleatum infection is prevalent in human colorectal carcinoma. Genome Res..

[149] Li Y.-Y., Ge Q.-X., Cao J., Zhou Y.-J., Du Y.-L., Shen B., Wan Y.-J.Y., Nie Y.-Q. (2016). Association of Fusobacterium nucleatum infection with colorectal cancer in Chinese patients. World J. Gastroenterol..

[150] Yang Y., Weng W., Peng J., Hong L., Yang L., Toiyama Y., Gao R., Liu M., Yin M., Pan C. (2017). Fusobacterium nucleatum Increases Proliferation of Colorectal Cancer Cells and Tumor Development in Mice by Activating Toll-Like Receptor 4 Signaling to Nuclear Factor-κB, and Up-regulating Expression of MicroRNA-21. Gastroenterology.

[151] Medina P.P., Nolde M., Slack F.J. (2010). OncomiR addiction in an in vivo model of microRNA-21-induced pre-B-cell lymphoma. Nature.

[152] Kostic A.D., Chun E., Robertson L., Glickman J.N., Gallini C.A., Michaud M., Clancy T.E., Chung D.C., Lochhead P., Hold G.L. (2013). Fusobacterium nucleatum potentiates intestinal tumorigenesis and modulates the tumor immune microenvironment. Cell Host Microbe.

[153] Rubinstein M.R., Wang X., Liu W., Hao Y., Cai G., Han Y.W. (2013). Fusobacterium nucleatum Promotes Colorectal Carcinogenesis by Modulating E-Cadherin/β-Catenin Signaling via its FadA Adhesin. Cell Host Microbe.

[154] McCOY W.C., Mason J.M. (1951). Enterococcal endocarditis associated with carcinoma of the sigmoid; report of a case. J. Med. Assoc. State Ala..

[155] Klein R.S., Recco R.A., Catalano M.T., Edberg S.C., Casey J.I., Steigbigel N.H. (1977). Association of *Streptococcus bovis* with carcinoma of the colon. N. Engl. J. Med..

[156] Boleij A., van Gelder M.M., Swinkels D.W., Tjalsma H. (2011). Clinical Importance of *Streptococcus gallolyticus* infection among colorectal cancer patients: Systematic review and meta-analysis. Clin. Infect. Dis..

[157] Corredoira-Sánchez J., García-Garrote F., Rabuñal R., López-Roses L., García-País M.J., Castro E., González-Soler R., Coira A., Pita J., López-Álvarez M.J. (2012). Association Between Bacteremia Due to *Streptococcus gallolyticus* subsp. *gallolyticus* (*Streptococcus bovis* I) and Colorectal Neoplasia: A Case-Control Study. Clin. Infect. Dis..

[158] Biarc J., Nguyen I.S., Pini A., Gossé F., Richert S., Thiersé D., Van Dorsselaer A., Leize-Wagner E., Raul F., Klein J.-P. (2004). Carcinogenic properties of proteins with pro-inflammatory activity from *Streptococcus infantarius* (formerly *S.bovis*). Carcinogenesis.

[159] Ellmerich S., Schöller M., Duranton B., Gossé F., Galluser M., Klein J.P., Raul F. (2000). Promotion of intestinal carcinogenesis by *Streptococcus bovis*. Carcinogenesis.

[160] Abdulamir A.S., Hafidh R.R., Bakar F.A. (2010). Molecular detection, quantification, and isolation of *Streptococcus gallolyticus* bacteria colonizing colorectal tumors: Inflammation-driven potential of carcinogenesis via IL-1, COX-2, and IL-8. Mol. Cancer.

[161] Ballard J., Bryant A., Stevens D., Tweten R.K. (1992). Purification and characterization of the lethal toxin (alpha-toxin) of *Clostridium septicum*. Infect. Immun..

[162] Kennedy C.L., Krejany E.O., Young L.F., O’Connor J.R., Awad M.M., Boyd R.L., Emmins J.J., Lyras D., Rood J.I. (2005). The alpha-toxin of *Clostridium septicum* is essential for virulence. Mol. Microbiol..

[163] Chew S.S., Lubowski D.Z. (2001). *Clostridium septicum* and malignancy. ANZ J. Surg..

[164] Corredoira J., Grau I., Garcia-Rodriguez J.F., García-País M.J., Rabuñal R., Ardanuy C., García-Garrote F., Coira A., Alonso M.P., Boleij A. (2017). Colorectal neoplasm in cases of *Clostridium septicum* and *Streptococcus gallolyticus* subsp. gallolyticus bacteraemia. Eur. J. Intern. Med..

[165] Mirza N.N., McCloud J.M., Cheetham M.J. (2009). *Clostridium septicum* sepsis and colorectal cancer—A reminder. World J. Surg. Oncol..

[166] Chakravorty A., Awad M.M., Cheung J.K., Hiscox T.J., Lyras D., Rood J.I. (2015). The Pore-Forming α-Toxin from *Clostridium septicum* Activates the MAPK Pathway in a Ras-c-Raf-Dependent and Independent Manner. Toxins.

[167] Suerbaum S., Michetti P. (2002). Helicobacter pylori infection. N. Engl. J. Med..

[168] Testerman T.L., Morris J. (2014). Beyond the stomach: An updated view of Helicobacter pylori pathogenesis, diagnosis, and treatment. World J. Gastroenterol..

[169] Meucci G., Tatarella M., Vecchi M., Ranzi M.L., Biguzzi E., Beccari G., Clerici E., de Franchis R. (1997). High prevalence of Helicobacter pylori infection in patients with colonic adenomas and carcinomas. J. Clin. Gastroenterol..

[170] Fujimori S., Kishida T., Kobayashi T., Sekita Y., Seo T., Nagata K., Tatsuguchi A., Gudis K., Yokoi K., Tanaka N. (2005). Helicobacter pylori infection increases the risk of colorectal adenoma and adenocarcinoma, especially in women. J. Gastroenterol..

[171] Hong S.N., Lee S.M., Kim J.H., Lee T.Y., Kim J.H., Choe W.H., Lee S.-Y., Cheon Y.K., Sung I.K., Park H.S. (2012). Helicobacter pylori infection increases the risk of colorectal adenomas: Cross-sectional study and meta-analysis. Dig. Dis. Sci..

[172] Inoue I., Mukoubayashi C., Yoshimura N., Niwa T., Deguchi H., Watanabe M., Enomoto S., Maekita T., Ueda K., Iguchi M. (2011). Elevated risk of colorectal adenoma with Helicobacter pylori-related chronic gastritis: A population-based case-control study. Int. J. Cancer.

[173] Siddheshwar R.K., Muhammad K.B., Gray J.C., Kelly S.B. (2001). Seroprevalence of helicobacter pylori in patients with colorectal polyps and colorectal carcinoma. Am. J. Gastroenterol..

[174] Bae R.C., Jeon S.W., Cho H.J., Jung M.K., Kweon Y.O., Kim S.K. (2009). Gastric dysplasia may be an independent risk factor of an advanced colorectal neoplasm. World J. Gastroenterol..

[175] Moss S.F., Neugut A.I., Garbowski G.C., Wang S., Treat M.R., Forde K.A. (1995). Helicobacter pylori seroprevalence and colorectal neoplasia: Evidence against an association. J. Natl. Cancer Inst..

[176] Kim T.J., Kim E.R., Chang D.K., Kim Y.-H., Baek S.-Y., Kim K., Hong S.N. (2017). Helicobacter pylori infection is an independent risk factor of early and advanced colorectal neoplasm. Helicobacter.

[177] Nam J.H., Hong C.W., Kim B.C., Shin A., Ryu K.H., Park B.J., Kim B., Sohn D.K., Han K.S., Kim J. (2017). Helicobacter pylori infection is an independent risk factor for colonic adenomatous neoplasms. Cancer Causes Control..

[178] Yan Y., Chen Y.-N., Zhao Q., Chen C., Lin C.-J., Jin Y., Pan S., Wu J.-S. (2017). Helicobacter pylori infection with intestinal metaplasia: An independent risk factor for colorectal adenomas. World J. Gastroenterol..

[179] Hartwich A., Konturek S., Pierzchalski P., Zuchowicz M., Labza H., Konturek P., Karczewska E., Bielanski W., Marlicz K., Starzynska T. (2001). Helicobacter pylori infection, gastrin, cyclooxygenase-2, and apoptosis in colorectal cancer. Int. J. Colorectal Dis..

[180] Koh T.J., Dockray G.J., Varro A., Cahill R.J., Dangler C.A., Fox J.G., Wang T.C. (1999). Overexpression of glycine-extended gastrin in transgenic mice results in increased colonic proliferation. J. Clin. Investig..

[181] Tatishchev S.F., VanBeek C., Wang H.L. (2012). Helicobacter pylori infection and colorectal carcinoma: Is there a causal association?. J. Gastrointest. Oncol..

[182] Handa O., Naito Y., Yoshikawa T. (2010). Helicobacter pylori: A ROS-inducing bacterial species in the stomach. Inflamm. Res..

[183] Shmuely H., Passaro D., Figer A., Niv Y., Pitlik S., Samra Z., Koren R., Yahav J. (2001). Relationship between Helicobacter pylori CagA status and colorectal cancer. Am. J. Gastroenterol..

[184] Cover T.L., Blanke S.R. (2005). Helicobacter pylori VacA, a paradigm for toxin multifunctionality. Nat. Rev. Microbiol..

[185] Evans D.J., Evans D.G., Takemura T., Nakano H., Lampert H.C., Graham D.Y., Granger D.N., Kvietys P.R. (1995). Characterization of a Helicobacter pylori neutrophil-activating protein. Infect. Immun..

[186] Wessler S., Krisch L.M., Elmer D.P., Aberger F. (2017). From inflammation to gastric cancer—The importance of Hedgehog/GLI signaling in Helicobacte r pylori-induced chronic inflammatory and neoplastic diseases. Cell Commun. Signal..

[187] Sousa C.P. (2006). The versatile strategies of Escherichia coli pathotypes: A mini review. J. Venom. Anim. Toxins Trop. Dis..

[188] Escobar-Páramo P., Grenet K., Le Menac’h A., Rode L., Salgado E., Amorin C., Gouriou S., Picard B., Rahimy M.C., Andremont A. (2004). Large-scale population structure of human commensal Escherichia coli isolates. Appl. Environ. Microbiol..

[189] Darfeuille-Michaud A., Neut C., Barnich N., Lederman E., Di Martino P., Desreumaux P., Gambiez L., Joly B., Cortot A., Colombel J.F. (1998). Presence of adherent Escherichia coli strains in ileal mucosa of patients with Crohn’s disease. Gastroenterology.

[190] Darfeuille-Michaud A., Boudeau J., Bulois P., Neut C., Glasser A.-L., Barnich N., Bringer M.-A., Swidsinski A., Beaugerie L., Colombel J.-F. (2004). High prevalence of adherent-invasive Escherichia coli associated with ileal mucosa in Crohn’s disease. Gastroenterology.

[191] Swidsinski A., Khilkin M., Kerjaschki D., Schreiber S., Ortner M., Weber J., Lochs H. (1998). Association between intraepithelial Escherichia coli and colorectal cancer. Gastroenterology.

[192] Martin H.M., Campbell B.J., Hart C.A., Mpofu C., Nayar M., Singh R., Englyst H., Williams H.F., Rhodes J.M. (2004). Enhanced Escherichia coli adherence and invasion in Crohn’s disease and colon cancer. Gastroenterology.

[193] Maddocks O.D.K., Short A.J., Donnenberg M.S., Bader S., Harrison D.J. (2009). Attaching and effacing Escherichia coli downregulate DNA mismatch repair protein in vitro and are associated with colorectal adenocarcinomas in humans. PLoS ONE.

[194] Buc E., Dubois D., Sauvanet P., Raisch J., Delmas J., Darfeuille-Michaud A., Pezet D., Bonnet R. (2013). High Prevalence of Mucosa-Associated *E. coli* Producing Cyclomodulin and Genotoxin in Colon Cancer. PLoS ONE.

[195] Prorok-Hamon M., Friswell M.K., Alswied A., Roberts C.L., Song F., Flanagan P.K., Knight P., Codling C., Marchesi J.R., Winstanley C. (2014). Colonic mucosa-associated diffusely adherent *afaC+ Escherichia coli expressing lpfA* and *pks* are increased in inflammatory bowel disease and colon cancer. Gut.

[196] Bonnet M., Buc E., Sauvanet P., Darcha C., Dubois D., Pereira B., Déchelotte P., Bonnet R., Pezet D., Darfeuille-Michaud A. (2014). Colonization of the human gut by *E. coli* and colorectal cancer risk. Clin. Cancer Res..

[197] Raisch J., Buc E., Bonnet M., Sauvanet P., Vazeille E., de Vallée A., Déchelotte P., Darcha C., Pezet D., Bonnet R. (2014). Colon cancer-associated B2 Escherichia coli colonize gut mucosa and promote cell proliferation. World J. Gastroenterol..

[198] Marchès O., Ledger T.N., Boury M., Ohara M., Tu X., Goffaux F., Mainil J., Rosenshine I., Sugai M., De Rycke J. (2003). Enteropathogenic and enterohaemorrhagic Escherichia coli deliver a novel effector called Cif, which blocks cell cycle G2/M transition. Mol. Microbiol..

[199] Flatau G., Lemichez E., Gauthier M., Chardin P., Paris S., Fiorentini C., Boquet P. (1997). Toxin-induced activation of the G protein p21 Rho by deamidation of glutamine. Nature.

[200] Taieb F., Petit C., Nougayrède J.-P., Oswald E. (2016). The Enterobacterial Genotoxins: Cytolethal Distending Toxin and Colibactin. EcoSal Plus.

[201] Nougayrède J.-P., Homburg S., Taieb F., Boury M., Brzuszkiewicz E., Gottschalk G., Buchrieser C., Hacker J., Dobrindt U., Oswald E. (2006). Escherichia coli induces DNA double-strand breaks in eukaryotic cells. Science.

[202] Cuevas-Ramos G., Petit C.R., Marcq I., Boury M., Oswald E., Nougayrède J.-P. (2010). Escherichia coli induces DNA damage in vivo and triggers genomic instability in mammalian cells. Proc. Natl. Acad. Sci. USA.

[203] Vizcaino M.I., Crawford J.M. (2015). The colibactin warhead crosslinks DNA. Nat. Chem..

[204] Secher T., Samba-Louaka A., Oswald E., Nougayrède J.-P. (2013). Escherichia coli producing colibactin triggers premature and transmissible senescence in mammalian cells. PLoS ONE.

[205] Cougnoux A., Dalmasso G., Martinez R., Buc E., Delmas J., Gibold L., Sauvanet P., Darcha C., Déchelotte P., Bonnet M. (2014). Bacterial genotoxin colibactin promotes colon tumour growth by inducing a senescence-associated secretory phenotype. Gut.

[206] Raisch J., Rolhion N., Dubois A., Darfeuille-Michaud A., Bringer M.-A. (2015). Intracellular colon cancer-associated Escherichia coli promote protumoral activities of human macrophages by inducing sustained COX-2 expression. Lab. Investig..

[207] Gagnière J., Bonnin V., Jarrousse A.-S., Cardamone E., Agus A., Uhrhammer N., Sauvanet P., Déchelotte P., Barnich N., Bonnet R. (2017). Interactions between microsatellite instability and human gut colonization by Escherichia coli in colorectal cancer. Clin. Sci. 1979.

[208] Tian Y., Wang K., Ji G. (2017). P112 Short-chain fatty acids administration is protective in colitis-associated colorectal cancer development. J. Crohns Colitis.

[209] Goodwin A.C., Destefano Shields C.E., Wu S., Huso D.L., Wu X., Murray-Stewart T.R., Hacker-Prietz A., Rabizadeh S., Woster P.M., Sears C.L. (2011). Polyamine catabolism contributes to enterotoxigenic Bacteroides fragilis-induced colon tumorigenesis. Proc. Natl. Acad. Sci. USA.

[210] Mauro M.O., Monreal M.T., Silva M.T., Pesarini J.R., Mantovani M.S., Ribeiro L.R., Dichi J.B., Carreira C.M., Oliveira R.J. (2013). Evaluation of the antimutagenic and anticarcinogenic effects of inulin in vivo. Genet. Mol. Res..

[211] Chen Z.-Y., Hsieh Y.-M., Huang C.-C., Tsai C.-C. (2017). Inhibitory Effects of Probiotic Lactobacillus on the Growth of Human Colonic Carcinoma Cell Line HT-29. Molecules.

[212] Del Carmen S., de Moreno de LeBlanc A., Levit R., Azevedo V., Langella P., Bermúdez-Humarán L.G., LeBlanc J.G. (2017). Anti-cancer effect of lactic acid bacteria expressing antioxidant enzymes or IL-10 in a colorectal cancer mouse model. Int. Immunopharmacol..

[213] Iida N., Dzutsev A., Stewart C.A., Smith L., Bouladoux N., Weingarten R.A., Molina D.A., Salcedo R., Back T., Cramer S. (2013). Commensal bacteria control cancer response to therapy by modulating the tumor microenvironment. Science.

[214] West N.R., Powrie F. (2015). Immunotherapy Not Working? Check Your Microbiota. Cancer Cell.

[215] Li T.-T., Ogino S., Qian Z.R. (2014). Toll-like receptor signaling in colorectal cancer: Carcinogenesis to cancer therapy. World J. Gastroenterol..

[216] Fukata M., Shang L., Santaolalla R., Sotolongo J., Pastorini C., España C., Ungaro R., Harpaz N., Cooper H.S., Elson G. (2011). Constitutive activation of epithelial TLR4 augments inflammatory responses to mucosal injury and drives colitis-associated tumorigenesis. Inflamm. Bowel Dis..

